# Urinary metabolic characterization of advanced tuberculous meningitis cases in a South African paediatric population

**DOI:** 10.3389/fmolb.2024.1253983

**Published:** 2024-03-15

**Authors:** Simon Isaiah, Du Toit Loots, Mari van Reenen, Regan Solomons, Sabine van Elsland, A. Marceline Tutu van Furth, Martijn van der Kuip, Shayne Mason

**Affiliations:** ^1^ Human Metabolomics, Faculty of Natural and Agricultural Sciences, North-West University, Potchefstroom, South Africa; ^2^ Department of Paediatrics and Child Health, Faculty of Medicine and Health Sciences, Stellenbosch University, Cape Town, South Africa; ^3^ MRC Centre for Global Infectious Disease Analysis, Imperial College London, London, United Kingdom; ^4^ Vrije Universiteit, Pediatric Infectious Diseases and Immunology, Amsterdam University Medical Centers, Emma Children’s Hospital, Amsterdam, Netherlands

**Keywords:** urine, metabolic, paediatric, tuberculous meningitis (TBM), untargeted metabolomics, Proton nuclear magnetic resonance (^1^H NMR) spectroscopy

## Abstract

Tuberculous meningitis (TBM) is a severe form of tuberculosis with high neuro-morbidity and mortality, especially among the paediatric population (aged ≤12 years). Little is known of the associated metabolic changes. This study aimed to identify characteristic metabolic markers that differentiate severe cases of paediatric TBM from controls, through non-invasive urine collection. Urine samples selected for this study were from two paediatric groups. Group 1: controls (n = 44): children without meningitis, no neurological symptoms and from the same geographical region as group 2. Group 2: TBM cases (n = 13): collected from paediatric patients that were admitted to Tygerberg Hospital in South Africa on the suspicion of TBM, mostly severely ill; with a later confirmation of TBM. Untargeted 1H NMR-based metabolomics data of urine were generated, followed by statistical analyses via MetaboAnalyst (v5.0), and the identification of important metabolites. Twenty nine urinary metabolites were identified as characteristic of advanced TBM and categorized in terms of six dysregulated metabolic pathways: 1) upregulated tryptophan catabolism linked to an altered vitamin B metabolism; 2) perturbation of amino acid metabolism; 3) increased energy production–metabolic burst; 4) disrupted gut microbiota metabolism; 5) ketoacidosis; 6) increased nitrogen excretion. We also provide original biological insights into this biosignature of urinary metabolites that can be used to characterize paediatric TBM patients in a South African cohort.

## 1 Introduction

The most lethal form of extra-pulmonary tuberculosis (TB)–tuberculous meningitis (TBM)–affects 1%–5% of TB infected individuals globally (Donovan et al., 2020). The pediatric age group (aged ≤12 years) are the most at risk for contracting the disease, and account for 12% of all TBM cases, with one in five affected children dying and only one in three surviving without long-term neurological sequelae (WHO, 2020; Basu Roy et al., 2021). According to the World Health Organization, South Africa is among the eight countries that account for two-thirds of the global total TB burden (WHO, 2020). In the Western Cape province of South Africa, TBM is the most common form of paediatric meningitis detected (Donald et al., 1996; Wolzak et al., 2012). Early diagnosis and timely introduction of appropriate therapy can potentiate a positive treatment outcome (Donovan et al., 2020). However, the timely and accurate diagnosis of TBM is challenging, since the early symptoms are usually nonspecific (van Toorn and Solomons, 2014).

There is a pressing need to improve current TBM diagnostic strategies (Hasbun et al., 2018). Stand-alone methods currently used to diagnose TBM in children are unreliable (Manyelo et al., 2021). Existing diagnostic techniques for TBM are invasive, complex and time-consuming, subsequently delaying treatment, and putting patients at a high risk of mortality. Recently, efforts have been made to better understand the pathophysiology through new research focused on finding novel TBM biomarker signatures (Rohlwink et al., 2017; Manyelo et al., 2019). Yet, there are still no clear biomarker(s), nor biosignature(s), for TBM in the cerebrospinal fluid (CSF), let alone one from patient samples collected non-invasively, such as from urine. Urine is rich in metabolic information that describes the systematic state of an individual and is a relatively unexplored biofluid in TBM research. Urinary metabolomics profiling in pulmonary TB patients has been an efficient means of diagnosing and monitoring an individual’s response to treatment (Luies et al., 2017). Mason *et al.* (2016b) holistically illustrated the metabolic complexity of TBM and provided proof-of-concept that a putative biosignature of urinary metabolites (methylcitric, 2-ketoglutaric, quinolinic and 4-hydroxyhippuric acids) can be defined with the potential to be used for the non-invasive metabolomics diagnosis and prognosis of paediatric TBM patients. In another metabolomics study, Chatterji *et al.* (2016) observed reduced malonic acid and elevated 2-hydroxybutyric acid, acetic acid, creatine and glycerophosphocholine, in the urine of TBM adults. These pioneering metabolomics studies have provided proof-of-concept that urine provides a wealth of metabolic information in TBM cases and deserves further investigation.

Metabolomics provides analytical, chemical and physiological insights into metabolite interactions (Mason et al., 2016a). These metabolites are vital constituents of biological systems and are highly informative of their functional state, serving as biomarkers of disease and also reflective of therapeutic response (Goodacre, 2010). van der Greef *et al.* (2004) describe metabolomics as a prospective tool for clinical use in the early detection of a metabolic perturbation in a biological system, before the disease symptoms actually present themselves. Nuclear magnetic resonance (NMR) spectroscopy is one such technique commonly used for the metabolite profiling and analysis of complex biofluids (Nagana Gowda et al., 2008). Studying the molecular level shift in equilibrium in patient urine will improve the systematic understanding of TBM and may also help develop transformable solutions related to novel diagnostics (Blankley et al., 2014). The need for research, such as that reported here, in order to generate knowledge which can be used to develop timely and accurate diagnosis, improve treatment outcomes, and ultimately reduce the dire mortality and neuro-morbidity in paediatric TBM disease in children (and adults), has become a priority (Organization, 2018). Hence, this study aimed to identify metabolites in urine that characterize the metabolic profile of severe TBM in paediatric cases, using ^1^H NMR metabolomics. Characterization here is defined as explaining the biochemistry underpinning TBM, in terms of altered metabolic pathways and increased/decreased metabolites.

## 2 Materials and methods

### 2.1 Sample origin and selection criteria

The sample population used in this study were infants and children (aged ≤12 years) from the Western Cape Province of South Africa, an area with high prevalence of TB (681 per 100,000), especially among children ≤12 years of age (100 per 966) (Donald et al., 1996; KANABUS, 2020). Participants were referred from primary and secondary level healthcare facilities to the paediatric department at Tygerberg Hospital, Stellenbosch University. The participants were divided into two groups (Group 1: control; n = 44) and (Group 2: TBM; n = 13). Criteria for collection of the controls (Group 1) were paediatric patients without meningitis, no neurological symptoms, and from the same geographical region as the TBM patients. Urine samples from the control group were requested from children undergoing routine follow up at Paediatric Outpatient Clinics, none of whom were acutely ill during sampling. The TBM urine samples (Group 2) used in this study were collected from pediatric patients (van Elsland et al., 2018) under initial treatment that were admitted to the hospital on suspicion of TBM, most of them severely sick; with a later confirmation of “definite TBM” according to the uniform research case definition for TBM (i.e., *M. tuberculosis* (*M.tb*) identified on CSF by microscopy, culture and/or detection by commercial nucleic-acid amplification test (Marais et al., 2010)) at an advanced stage (with or without focal neurological deficit) (Van Toorn et al., 2012). The urine sample used in this study was the first urine sample collected as an out-patient (discharged from hospital once stabilized) as a home-treatment program (van Elsland et al., 2018). Clinical information was recorded for all TBM cases (see Table 1). As per ethics requirements, all urine samples were collected after written informed consent from parent(s) and assent from the child, if older than 7 years and able to do so, was obtained, under the Health Research Ethics Committee (HREC) approval of Stellenbosch University (ethics approval no. N16/11/142 and N11/03/061 for group 1 and 2 respectively), the Western Cape provincial government, as well as by the HREC of the North-West University, Potchefstroom campus (ethics approval no. NWU-00063-18-A1-01). Participants with an unknown or positive HIV status were excluded from this study because HIV co-infection further confounds an already complex metabolic profile.

**TABLE 1:** Demographic, clinical, laboratory and imaging findings of 13 paediatric patients diagnosed with advanced tuberculous meningitis.

CRITERIA	n (%)
Gender: male/female	6 (46.2)/7 (53.8)^a^
Age (months) (Median [range])	43 [22–140]
Clinical symptoms	
Fever	11/13 (84.6)
Night sweats	2/13 (15.4)
Poor feeding	5/13 (38.5)
Weight loss	5/13 (38.5)
Vomiting	4/13 (30.8)
Coughing	None
Headache	1/13 (7.7)
Seizures	4/13 (30.8)
Lethargy	2/13 (15.4)
Neurological signs	
GCS: (median [range])	10 [7–15]
Meningism	3/13 (23.1)
Focal motor deficit	4/13 (30.8)
Cranial nerve palsy	3/13 (23.1)
Raised ICP	4/13 (30.8)
Neuroimaging (CT brain)	
Hydrocephalus	11/13 (84.6)
Infarctions	1/13 (7.7)
Tuberculoma	2/13 (15.4)
Meningeal enhancement	7/13 (53.8)
VP shunt	4/13 (30.8)
Convulsions	1/13 (7.7)
Hemiparesis	4/13 (30.8)
CXR (signs of pulmonary TB)	1/13 (7.7)
Laboratory values	
Blood albumin (g/L) (median [range])	41 [40–52]
Blood sodium (mmol/L) (median [range])	131.5 [120–141]
Blood total protein (g/L) (median [range])	77.5 [75–80]
Blood glucose (mmol/L) (median [range])	7.1 [4.9–7.7]
Blood lipids (median)	3.9
CSF protein (g/L) (median [range])	0.84 [0.21–3.0]
CSF glucose (mmol/L) (median [range])	2.2 [0.2–4.8]
CSF pleocytosis (cells/µL) (median [range])	40 [3–129]
CSF lymphocytes (cells/µL) (median [range])	57 [3–148])
BCG positive status	3/13 (23.1)

^a^ = so significant difference; ICP = intracranial pressure; VP = ventriculoperitoneal; BCG = *Bacillus* Calmette–Guérin vaccine; GCS = glasgow coma scale; CSF = cerebrospinal fluid; CXR = chest X-ray.

### 2.2 Sample transport, storage and handling

Upon collection, urine samples were stored at −80°C in a dedicated freezer at the division of Molecular Biology and Human Genetics at Stellenbosch University. Once all samples were collected, they were collectively couriered overnight–frozen, on dry ice, to a dedicated freezer (−80°C) in a biosafety level 3 (BSL3) laboratory at the Centre for Human Metabolomics, situated on the Potchefstroom campus of North-West University. All urine samples were thawed in a biological safety cabinet, after which 600 µL of each sample was aliquoted into a separate tube for NMR analysis and an additional 50 µL for the purpose of making a pooled quality control (QC) sample. The pooled QC sample was then vortexed and re-aliquoted into 20 equal-volume quantities. All aliquoted samples (including QC samples) were kept at −80°C until NMR analysis.

### 2.3 Sample preparation and ^1^H NMR analysis

All urine samples were thawed to room temperature prior to processing. A volume of 600 µL of urine was centrifuged at 12,000 *g* for 5 min to sediment any particulates and macromolecules. A volume of 540 µL of the supernatant was collected in a micro-centrifuge tube, with 60 µL of the NMR buffer solution [1.5 M potassium phosphate solution in deuterium oxide with internal standard TSP (trimethylsilyl-2,2,3,3-tetradeuteropropionic acid); pH 7.4]. The sample was briefly vortexed to ensure homogeneity before being centrifuged at 12,000 *g* for 5 min. For ^1^H NMR analysis, 540 µL of the supernatant was transferred to a 5 mm NMR glass tube and analyzed using a Bruker Avance III HD NMR spectrometer with a triple-resonance inverse (TXI) ^1^H (^15^N,^13^C) probe head and x, y, and z gradient coils, at 500 MHz, in a randomized sequence, with QC samples interweaved at regular intervals. With a spectral width of 12,000 Hz, ^1^H spectra were recorded as 128 transients in 32 K data points. The sample temperature was kept constant at 300 K, and the H2O resonance was pre-saturated using single-frequency irradiation with a 4-s relaxation delay and an 8-µs excitation pulse, using the noesygppr1d water presaturation pulse program. Sample shimming was performed automatically based on the deuterium signal. TSP and metabolites had resonance line widths of <1 Hz. Fourier transformation, phase and baseline correction were performed automatically. Bruker Topspin (V3.5) was used to process the NMR data. For metabolite identification and quantification, Bruker AMIX (V3.9.14) was employed (Erasmus et al., 2019).

### 2.4 Data pre-processing and statistical analysis

**FIGURE 1:**
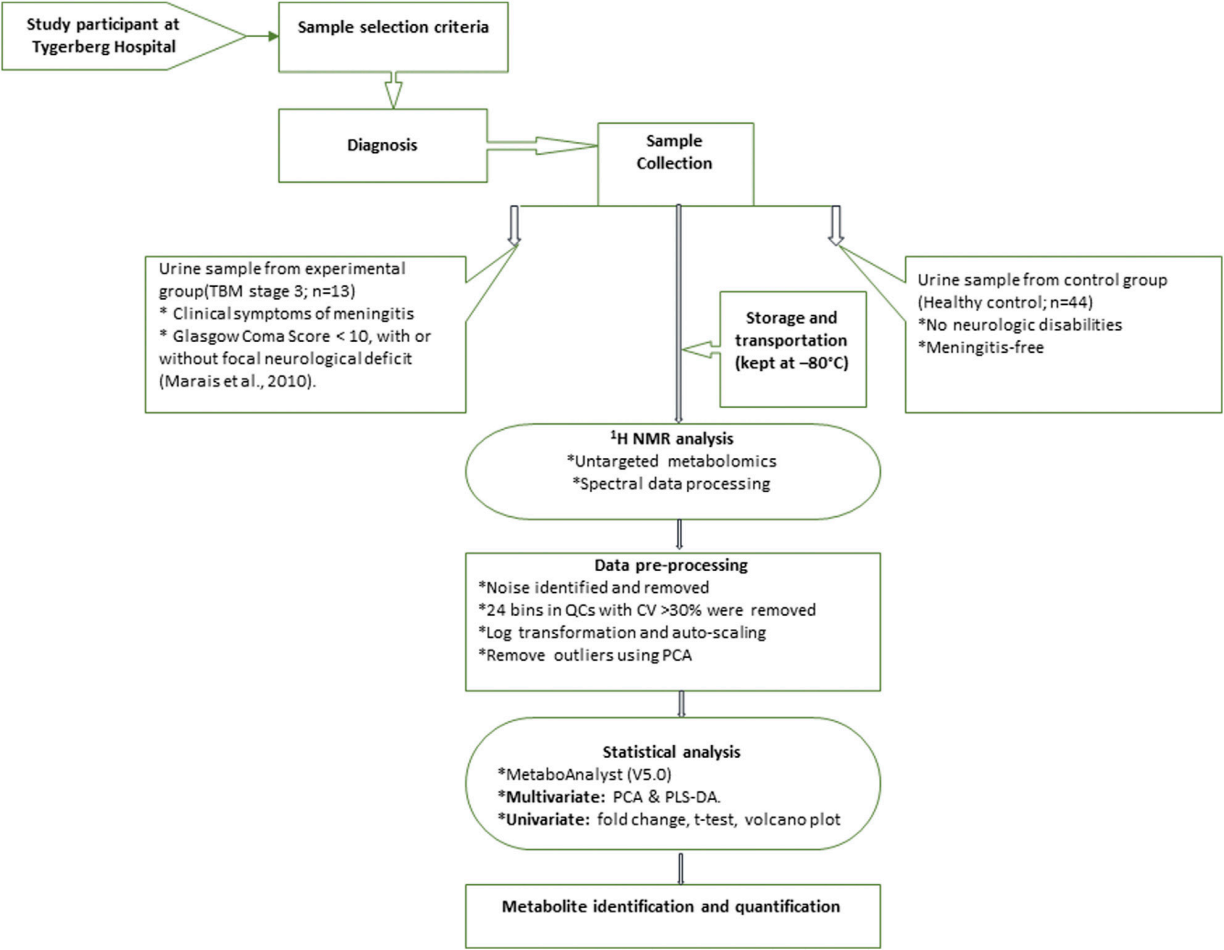
Schematic diagram indicating sequence of steps of experimental design of ^1^H NMR metabolomics and statistical analyses performed.

The ^1^H NMR spectral output was binned at 0.02 ppm widths, relative to creatinine, to create a data matrix of spectral intensity with the bins as the columns and samples as the rows. A t-test on the peak integral region of the creatinine peak between the control and TBM group revealed a *p*-value of 0.065. The noise level was identified as less than the limit of detection (LOD), calculated as: LOD = average of blank bins (bins with no discernible peaks) + 3.3 * standard deviation of blank bins (Westgard, 2008), and bins identified as noise were zeroed. All zeroes were replaced with 1/5 of the lowest value using MetaboAnalyst (V5.0). Bins around the suppressed water peak ∼4.72 ppm were also removed. The final data matrix gave a total of 468 bins. Throughout the batch analysis, 20 aliquots of a pooled QC sample were processed at predefined intervals to ensure trustworthy data. Quality assurance was based on QC observations, with bins with a coefficient of variation (CV) value greater than 30% were removed from the spectral binned data matrix. Because spectral intensity does not always reflect biological significance, data were log transformed and Pareto scaled to account for skewed distributions and to put bins on an even footing when presented for multivariate analysis. Statistical analyses and identification of important metabolites were conducted using MetaboAnalyst (V5.0)–a comprehensive platform dedicated for metabolomics data analysis via a web-based interface that enables high-throughput analysis for both targeted and untargeted metabolomics (Pang et al., 2021); included univariate statistics (fold changes and t-tests, displayed using a volcano plot) and multivariate statistics, specifically principal component analysis (PCA), partial least squares–discriminant analysis (PLS-DA). To project the data onto fewer, more manageable dimensions and to highlight entirely data-driven connections between instances, unsupervised PCA analyses were used with a 95% confidence interval (CI) ellipsis to detect natural separation between group centroid, to remove outliers in the two experimental groups, and to examine QC distribution to assess overall method reliability. Hierarchical cluster analysis was also performed, based on Euclidean distance using Ward’s linkage method. Quantitative statistical data were used to identify variables of importance: PLS-DA VIP of >1.0 for components 1 and 2, a t-test *p*-value ≤0.05, corrected for multiple testing (FDR, false discovery rate), and a fold change ≥2.0. Discriminatory metabolites were identified using pure compound 1D ^1^H NMR spectral libraries and confirmed using 2D correlation spectroscopy (COSY) and J-resolved (JRES) ^1^H–^1^H NMR data. Important identified metabolites were quantified relative to the creatinine peak (µmol/mmol creatinine) and additional univariate measures, including t-test *p*-values (adjusted using Bonferroni–Holm) and Cohen’s d-values, were calculated. Statistical significance (*p* ≤ 0.05) was used to generalize findings, that is, what is the probability that we won't find a difference if we take another sample? On the other hand, practical significance (d > 0.6) indicates magnitude of difference and answers the “so what” question, namely, whether the effect is large enough to care about. A summary of the experimental design for this study is illustrated schematically in Figure 1. The use of online metabolite databases, such as the Human Metabolome Database (HMDB) and the Kyoto Encyclopedia of Genes and Genomes (KEGG), were used to assist in the biological interpretation of the results.

## 3 Results

**FIGURE 2:**
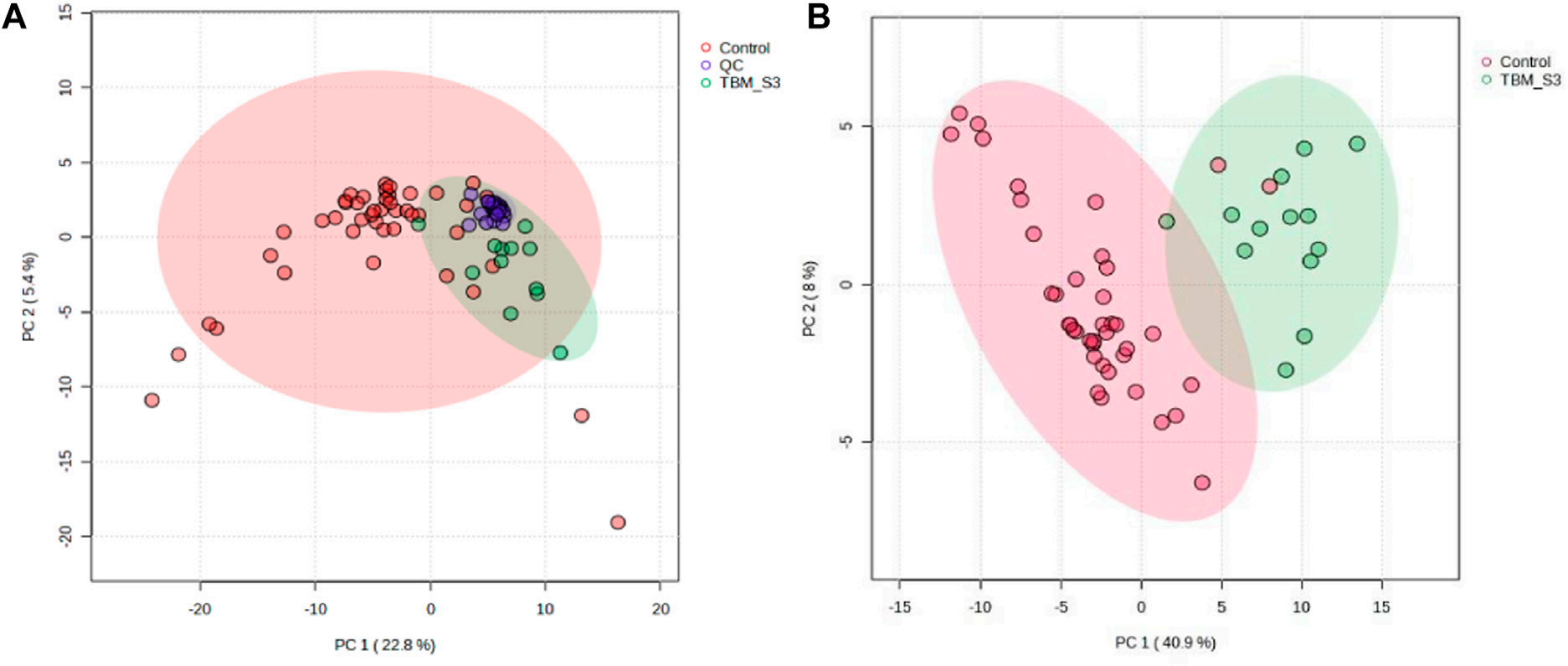
PCA scores plots. **(A)** (Left): Quality controls (QCs) and all cases–20 QCs, 44 controls and 13 TBM cases at initial treatment (TBM), with 95% CI ellipses. The total explained variance from PC1 and PC2 is 28.2%. **(B)** (Right): Reduced cases (without QCs)–40 controls and 13 TBM cases at initial treatment (TBM), with 95% CI ellipses. The total explained variance from PC1 and PC2 is 48.9%.

Prior to statistical analysis, a quantitative quality assurance check was performed on all QC samples. A total of 24 bins across 20 QC samples had a CV greater than 30% and were removed from the spectral binned data matrix. This reduced the number of bins from 468 to 444. Hence, the variation exhibited across bins between the two groups can be attributed towards biological variation. A PCA of all cases, including the QC samples (Figure 2A), shows that the 20 QC samples cluster closely together, indicating overall method reliability (i.e., no batch or machine drift). An unsupervised PCA, excluding QC samples, was performed Supplementary Figure S1 and four outliers were identified in the control group and removed from further analysis; no outliers identified in the TBM group. Next, an unsupervised PCA analysis of the 40 controls against the 13 TBM cases was performed (Figure 2B), which yielded almost complete natural separation between the groups, with their 95% CI ellipses slightly overlapping as a result of two control samples. The results of the unsupervised PCA confirm that the two groups are indeed differentiated, providing confidence before using the supervised method of PLS-DA.

Subsequent hierarchical cluster analysis (Supplementary Figure S2) identified two clusters: one homogeneous cluster containing control cases (n = 38) at the top, with one TBM case, and the other (bottom) cluster containing 12 TBM cases and two control cases. The hierarchical cluster analysis further illustrates that there was some overlap between the two groups, but overall there was differentiation.

**FIGURE 3:**
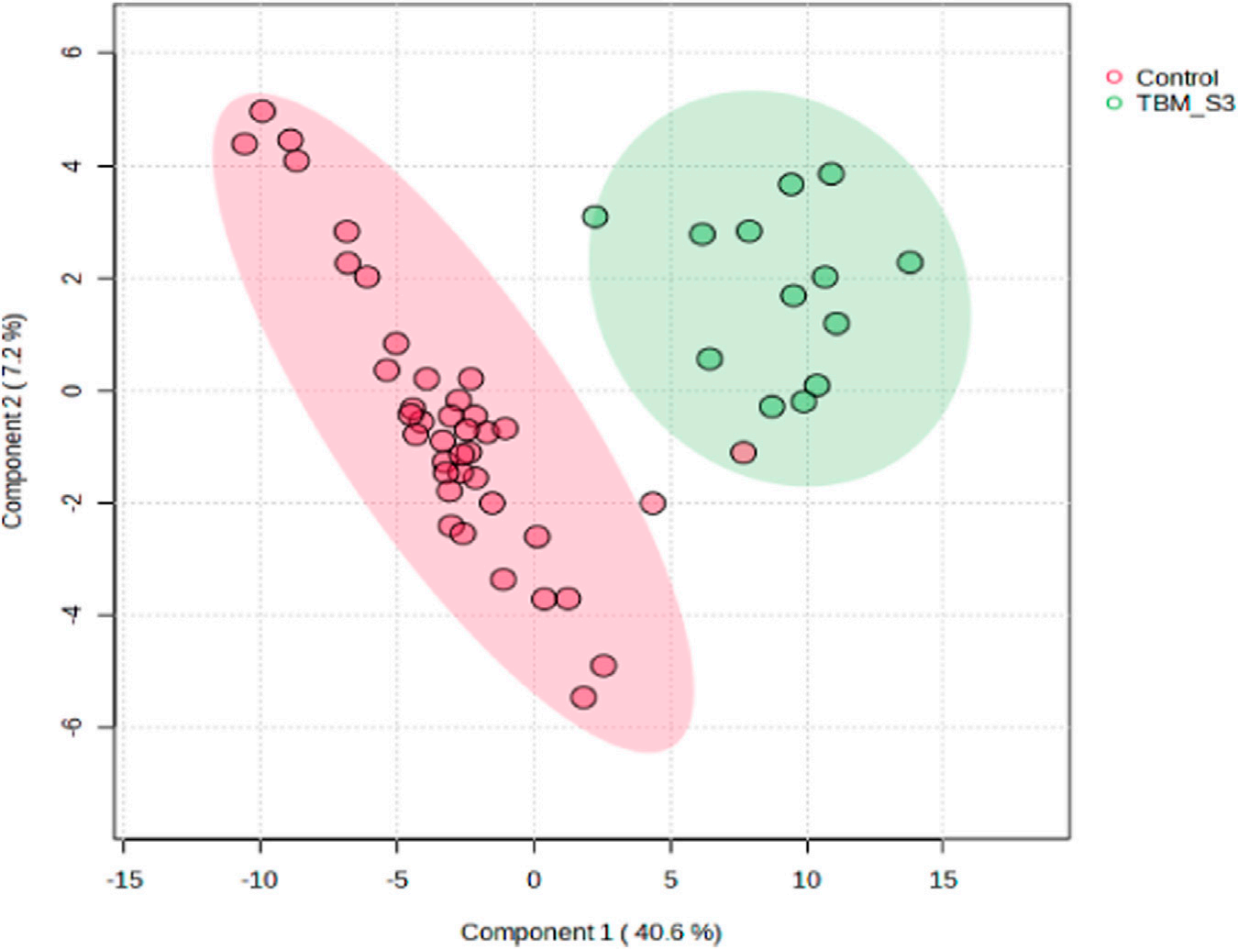
Supervised PLS-DA scores plot of 40 controls and 13 TBM cases at initial treatment (TBM), with 95% CI ellipses. The total explained variance from component 1 and component 2 is 47.8%.

A supervised PLS-DA (Figure 3) was performed in order to detect differentiating bins and avoid false discoveries. The PLS-DA model generates meaningful information that can be used to identify important variables and assess the significance of class discrimination by performing a permutation test using the optimal number of components determined by cross validation. The supervised PLS-DA model for the data exhibited an R^2^ of 96%, and was validated by a Q^2^ of 81%, and a permutation *p*-value of 0.0004 (188/2000). Various bins differentiating the TBM and control groups were observed through loading plots for the PLS-DA and identified through a variable of importance in projection (VIPs) cut-off criteria of greater than 1.0 (VIP >1.0) for components 1 and 2.

**FIGURE 4:**
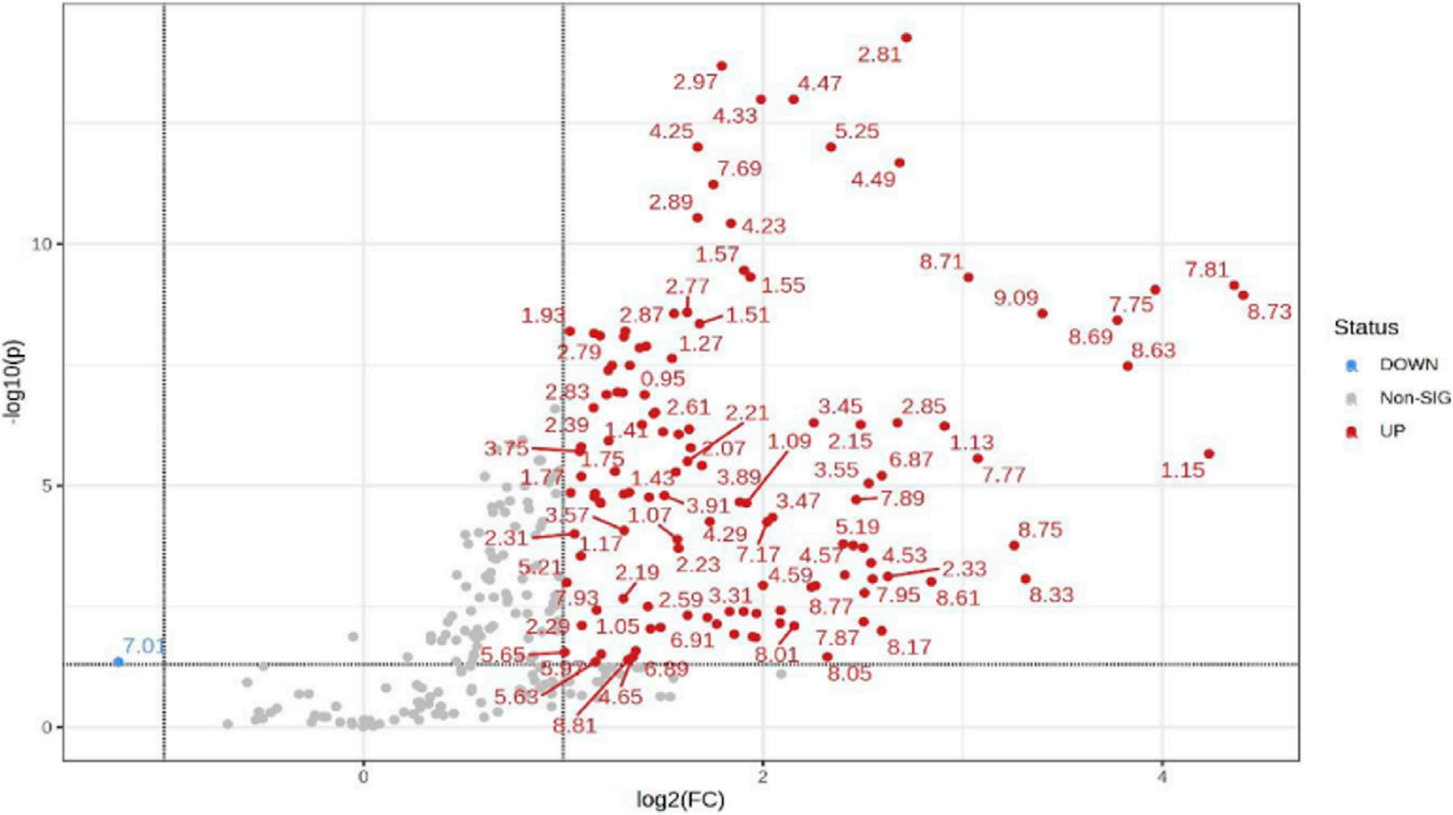
Volcano plot showing univariate statistically significant bins. Thresholds of absolute fold change ≥2.0 (log2) and *p*-value ≤0.05 (log10) are given as dotted lines. The red circles represent features above the threshold–notably, almost all significant bins are increased in TBM cases. Note that both fold changes and *p*-values are log transformed on the axes.

Additionally, univariate statistics of fold change analysis and Wilcoxon t-tests are illustrated as a volcano plot in (Figure 4). Thresholds of absolute fold change ≥2.0 and an FDR *p*-value ≤0.05 were used to identify statistically significant bins.

**FIGURE 5:**
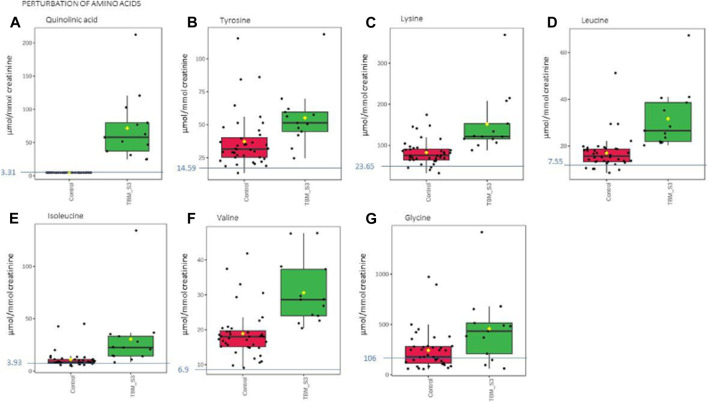
Box plots of the relative concentration (µmol/mmol creatinine) of seven urinary metabolites **(A)** quinolinic acid, **(B)** tyrosine, **(C)** lysine, **(D)** leucine, **(E)** isoleucine, **(F)** valine, **(G)** glycine] identified as being part of perturbation of amino acid metabolism in TBM. Green = TBM cases; red = controls. Blue line (and number) indicates normal reference value, based upon www.hmdb.ca and Bouatra *et al.* (2013).

**FIGURE 6:**
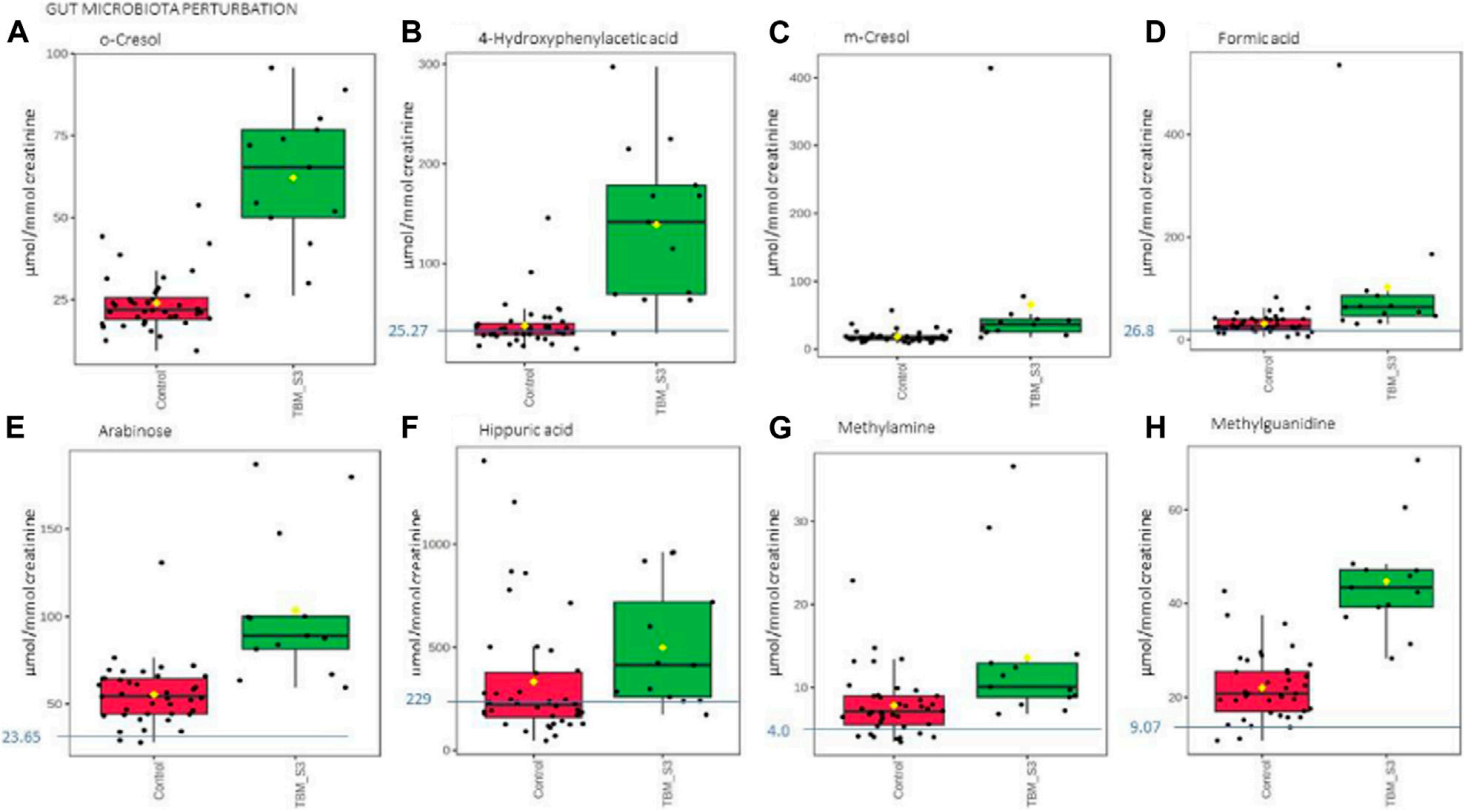
Box plots of the relative concentration (µmol/mmol creatinine) of eight urinary metabolites **(A)** o-cresol, **(B)** 4-hydroxyphenylacetic acid, **(C)** m-cresol, **(D)** formic acid, **(E)** arabinose, **(F)** hippuric acid, **(G)** methylamine, **(H)** methylguanidine] identified as being part of gut microbiota perturbation in TBM. Green = TBM cases; red = controls. Blue line (and number) indicates normal reference value, based upon www.hmdb.ca and Bouatra *et al.* (2013).

**FIGURE 7:**
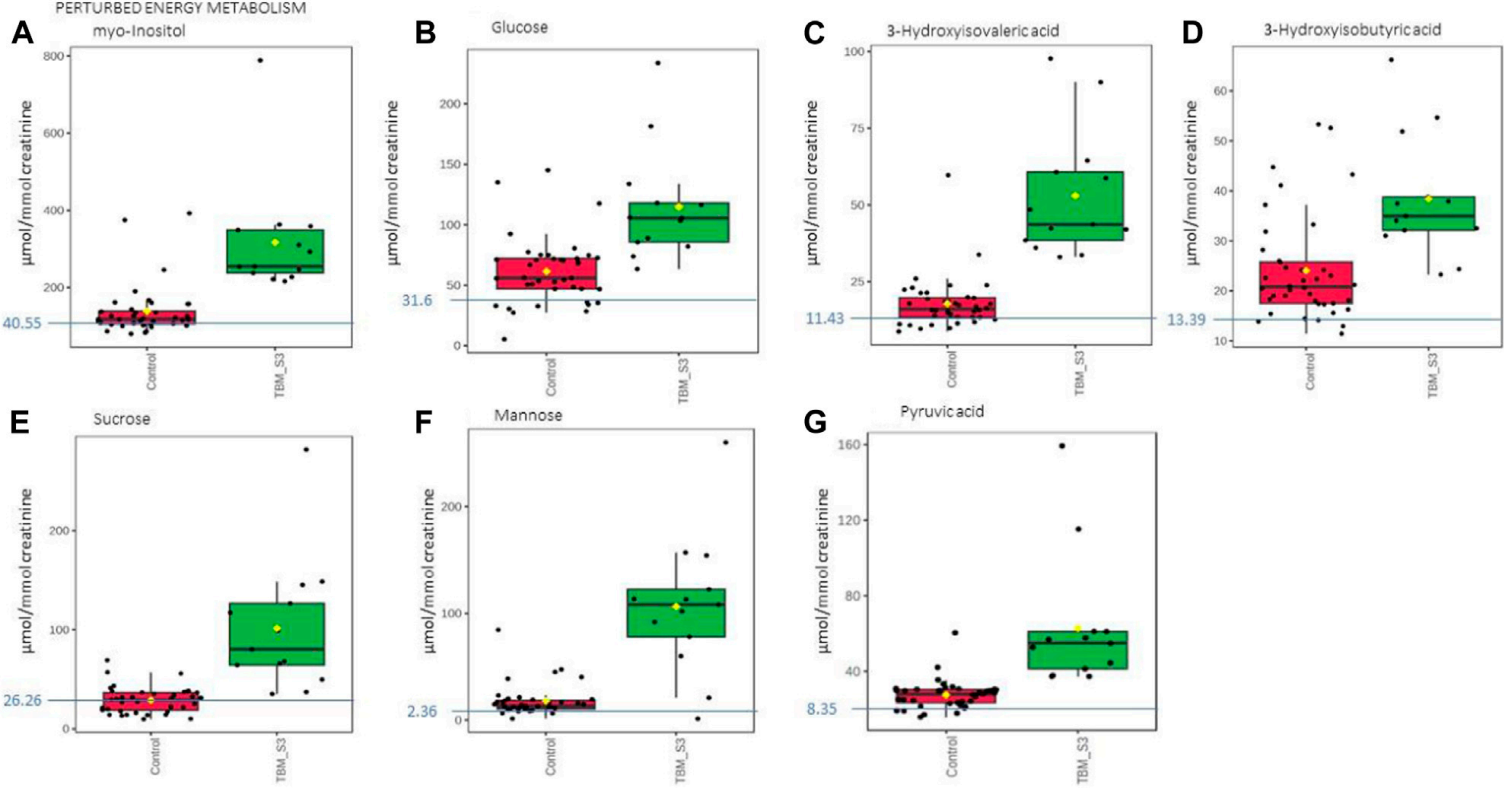
Box plots of the relative concentration (µmol/mmol creatinine) of seven urinary metabolites **(A)** myo-inositol, **(B)** glucose, **(C)** 3-hydroxyisovaleric acid. **(D)** 3-hydroxyisobutyric acid, **(E)** sucrose, **(F)** mannose, **(G)** pyruvic acid] identified as being part of perturbed energy metabolism in TBM. Green = TBM cases; red = controls. Blue line (and number) indicates normal reference value, based upon www.hmdb.ca and Bouatra *et al.* (2013).

**FIGURE 8:**
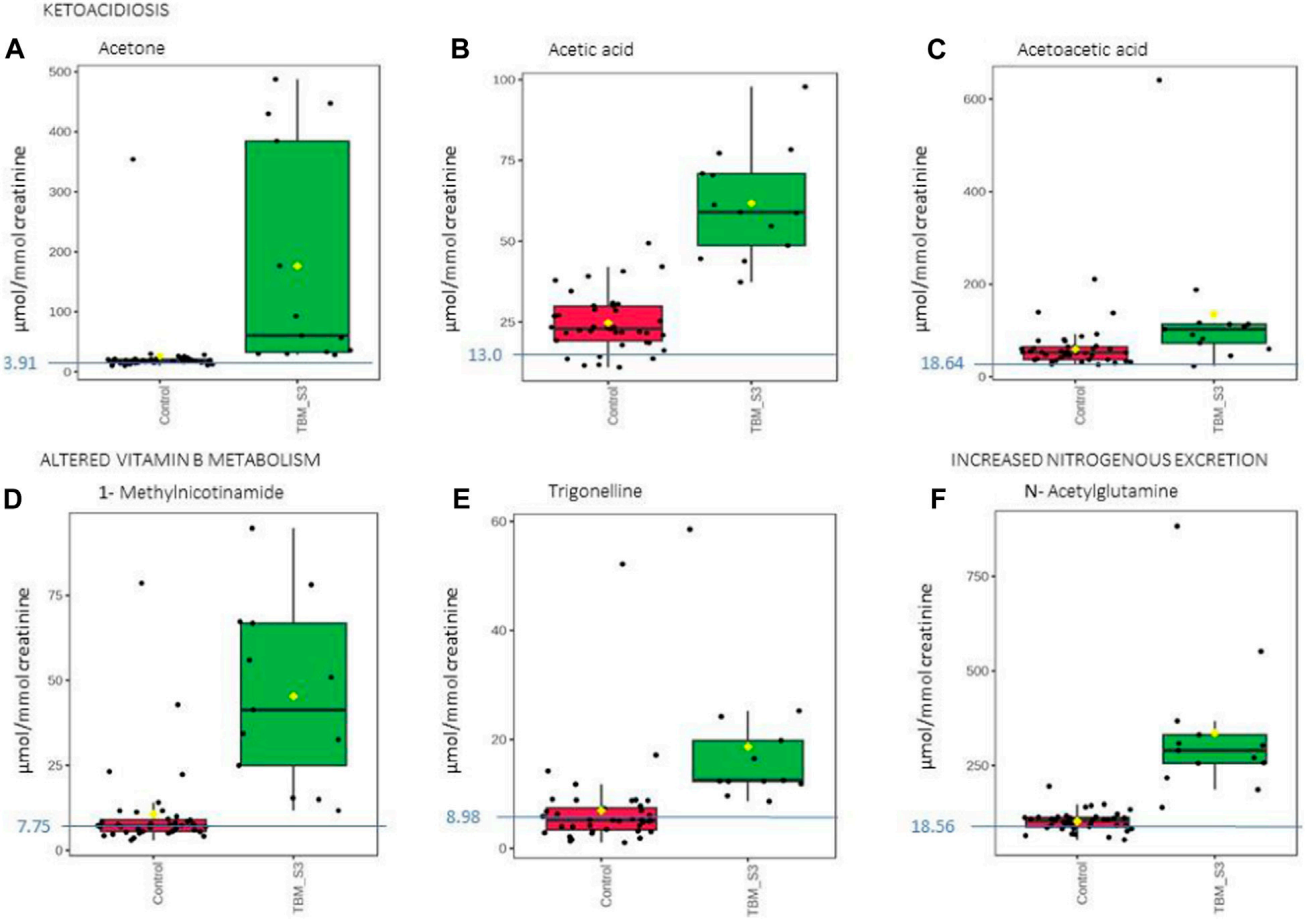
Box plots of the relative concentration (µmol/mmol creatinine) of six urinary metabolites identified as being part of ketoacidosis **(A)** acetone, **(B)** acetic acid, **(C)** acetoacetic acid], altered vitamin B metabolism **(D)** 1-methylnicotinamide, **(E)** trigonelline] and increased nitrogen excretion **(F)** N- acetylglutamine] in TBM. Green = TBM cases; red = controls. Blue line (and number) indicates normal reference value, based upon www.hmdb.ca and Bouatra *et al.* (2013).

Based upon the quantitative statistical data of the ^1^H-NMR spectral bins, the important bins were identified using the following rule: (VIP comp one and comp 2 > 1.0) OR (*p*-value FDR ≤0.05 and absolute fold change ≥2.0) (see Supplementary Table S1). Pure compound ^1^H NMR spectral libraries were used to annotate most of the important bins that discriminated between the 40 controls and the 13 cases of TBM at initial treatment. Several important bins contained either pure forms of medications, or their metabolites: 1,2-propanediol, acetaminophen, isoniazid, isonicotinic acid, acetylisoniazid, pyrazine carboxamide, pyrazine carboxylic acid, 2-pyridin-4-formidoacetic acid, 5-hydroxy-2-pyrazine carboxylic acid. 1,2-Propanediol (or propylene glycol) is used as a solvent for the preparation of pharmaceuticals and is a common vehicle for some paediatric medication (Komoroski et al., 2000). Aspirin, or acetylsalicylic acid, is a medication in the family of salicylates and the derivative salicyluric acid (2-hydroxyhippuric acid) is the glycine conjugation product (Erasmus et al., 2019). Isoniazid–a first-line TB treatment drug, was identified, along with two of its metabolites–isonicotinic acid, acetylisoniazid. Four metabolites (pyrazine carboxamide, pyrazine carboxylic acid, 2-pyridin-4-formidoacetic acid, 5-hydroxy-2-pyrazine carboxylic acid) of the first-line TB drug pyrazinamide were also identified. These medications are excluded from our discussion because they are of exogenous origin. A total of 29 metabolites were identified that characterize the urinary metabolic profile of the TBM cases in this cohort. The relative concentrations (µmol/mmol creatinine) of 28 metabolites were calculated (Figures 5–8) and the *p*-value and effects size were determined (Table 2). The resulting 28 metabolites were classified as: perturbed amino acid metabolism Figure 5: a) quinolinic acid, b) tyrosine, c) leucine, d) 3-hydroxyisobutyric acid, e) lysine, f) isoleucine, g) valine, h) glycine]; gut microbiota perturbation Figure 6: a) o-cresol, b) 4-hydroxyphenylacetic acid, c) m-cresol, d) formic acid, e) arabinose, f) hippuric acid, g) methylamine, h) methylguanidine]; perturbed energy metabolism Figure 7: a) myo-inositol, b) 3-hydroxyisovaleric acid, c) glucose, d) sucrose, e) mannose, f) pyruvic acid]; ketoacidosis [Figure 8: a) acetone, b) acetic acid, c) acetoacetic acid]; altered vitamin B metabolism Figure 8: d) 1-methylnicotinamide, e) trigonelline]; increased nitrogen excretion Figure 8: f) N-acetylglutamine, as well as increased urea–not quantified].

**TABLE 2:** Data of the quantified (concentration: µmol/mmol creatinine) important metabolites (n = 28) that characterize TBM. A *p*-value ≤0.05 indicates statistical significance, and a Cohen’s d effect size ≥0.6 indicates practical significance.

Metabolites (chemical shift)	HMDB ID	*p*-value	Effect size
1-Methylnicotinamide (9.29)	HMDB0000699	<0.001	1.82
3-Hydroxyisobutyric acid (1.08)	HMDB0000023	0.001	1.3
3-Hydroxyisovaleric acid (1.27)	HMDB0000754	<0.001	2.48
4-Hydroxyphenylacetic acid (6.88)	HMDB0000020	<0.001	2.07
Acetic acid (1.92)	HMDB0000042	<0.001	2.97
Acetoacetic acid (2.29)	HMDB0000060	0.111	0.81
Acetone (2.33)	HMDB0001659	0.014	1.29
Arabinose (4.54)	HMDB0000646	0.001	1.67
Formic acid (8.46)	HMDB0000142	0.08	0.99
Glucose (4.67)	HMDB0000122	0.001	1.48
Glycine (3.57)	HMDB0000123	0.053	0.81
Hippuric acid (7.66)	HMDB0000714	0.096	0.57
Isoleucine (1.03)	HMDB0000172	0.052	0.99
Leucine (0.99)	HMDB0000687	0.002	1.49
Lysine (1.76)	HMDB0000182	0.007	1.34
Mannose (5.20)	HMDB0000169	<0.001	2.3
m-Cresol (2.31)	HMDB0002048	0.135	0.85
Methylamine (2.60)	HMDB0000164	0.043	0.93
Methylguanidine (2.83)	HMDB0001522	<0.001	2.56
myo-Inositol (4.09)	HMDB0000211	0.001	1.71
N-Acetylglutamine (2.08)	HMDB0006029	<0.001	2.21
o-Cresol (2.21)	HMDB0002055	<0.001	2.58
Pyruvic acid (2.39)	HMDB0000243	0.004	1.70
Quinolinic acid (8.03)	HMDB0000232	<0.001	2.9
Sucrose (5.42)	HMDB0000258	0.002	1.89
Trigonelline (9.13)	HMDB0000875	0.008	1.14
Tyrosine (6.91)	HMDB0000158	0.021	0.85
Valine (1.05)	HMDB0000687	<0.001	1.48

Note: chemical shifts (ppm) of metabolites are given in brackets. HMDB ID refers to the identity number assigned to the metabolite in the Human Metabolome Database (www.hmdb.ca).

## 4 Discussion

The 29 significant urinary metabolites identified in this study can be categorized in terms of six dysregulated metabolic pathways. 1) Upregulated tryptophan catabolism (quinolinic acid) linked to an altered vitamin B3 metabolism (1-methylnicotinamide, trigonelline). 2) Perturbation of amino acid metabolism (leucine, lysine, isoleucine, glycine, tyrosine, valine). 3) Increased energy production–metabolic burst (3-hydroxyisobutyric acid, 3-hydroxyisovaleric acid, glucose, mannose, myo-inositol, pyruvic acid, sucrose). 4) Disrupted gut microbiota metabolism (4-hydroxyphenylacetic acid, arabinose, formic acid, hippuric acid, m-cresol, methylamine, methylguanidine, o-cresol). 5) Ketoacidosis (acetic acid, acetoacetic acid, acetone). 6) Increased nitrogen excretion (urea, N- acetylglutamine). We provide biological context by describing the characterization of the urinary metabolic profile of TBM in the discussion below.

### 4.1 Upregulated tryptophan catabolism

Quinolinic acid (Figure 5A) was significantly elevated in the urine of the TBM patients (71.57 ± 49.42 μmol/mmol creatinine) compared to the controls (0 μmol/mmol creatinine; *p* < 0.001, d = 2.9). Quinolinic acid was completely absent (or below the detection limit of the NMR spectrometer) in all control cases. This suggests that quinolinic acid would be an excellent candidate as a potential urinary diagnostic marker for TBM–to be tested in a future study.

Tryptophan is a key amino acid required for protein biosynthesis, and a precursor for the synthesis of a diversity of other metabolites (Davis et al., 2019); the most reported on, in the case of TB (Campbell et al., 2014; Manyelo et al., 2019), is the kynurenine pathway, via the *M. tb* infection-induced pro-inflammatory cytokines IL-6, TNF-α, and IFN-γ, upregulating indoleamine 2,3-dioxygenase (IDO) (Campbell et al., 2014). Quinolinic acid (a downstream metabolite of tryptophan), induced via IDO-1 (Heyes et al., 1992), has been associated with a variety of inflammatory disorders, elevated, and also detected in elevated concentrations in the CSF and brain tissue of patients with a wide range of infectious and other neurological diseases (Heyes et al., 2001), produced in large quantities by activated macrophages and microglia (Heyes et al., 1996). Of note, a study on the CSF collected from adult TBM cases done by van Laarhoven *et al.* (2018) identified tryptophan metabolism as one of the most upregulated metabolic pathways in TBM cases, with downstream upregulated tryptophan metabolism reflected by the kynurenine pathway. Their study examined an adult cohort of 33 TBM cases against 22 controls; with a further validation study of 101 TBM cases verses 17 controls. Of the metabolites identified in the initial study, 250 metabolites were increased and 18 decreased in TBM. Van Laarhoven *et al.* further examined the initial 33 TBM cases and identified 16 as survivors and 17 as non-survivors; 13 metabolites were shown to be increased in TBM survivors compared to controls and even higher in TBM non-survivors compared to TBM survivors. Of the 13 metabolites identified, three metabolites, including tryptophan, were found to be decreased in non-survivors compared to controls, and also decreased in TBM survivors *versus* TBM non-survivors. Van Laarhoven *et al.* then carried out a validation study on CSF tryptophan with 101 TBM cases *versus* 17 controls; that identified IDO-1 as having greater expression in TBM. However, in all of these investigation studies, there is no report of the metabolites that characterize TBM. In a study characterizing the CSF immunological signature of TBM in a cohort of 23 children (Manyelo et al., 2019), a 3-marker signature associated with neuroinflammation (VEGF, IFN-*γ*, and MPO) showed strong potential as a diagnostic tool for TBM in children with promising accuracy. We modelled the downstream metabolic effects expected from VEGF, IFN-*γ*, and MPO and predicted pivotal altered metabolic pathways that would be reflected in the urinary profiles of TBM subjects (Isaiah et al., 2020). Quinolinic acid was one of the major metabolic end-products that we predicted to come from this, within the *M. tb*-infected brain, induced by an increased IFN-*γ*. Urinary quinolinic acid was also observed in the TBM patients’ urine samples in a similar untargeted urinary ^1^H NMR metabolomics study carried out by Mason *et al.* (2016b). Quinolinic acid is not only expected in abundance in the *M. tb*-infected brain, but also produced by the enteric nervous system in the gut caused by dysbiosis, in conjunction with a perturbed gut–brain axis (Isaiah et al., 2020).

### 4.2 Altered vitamin B3 metabolism

Trigonelline (1-methylnicotinic acid) was significantly elevated (Figure 8E) in the urine of the TBM patients (18.65 ± 12.58 μmol/mmol creatinine) when compared to that of the controls (6.94 ± 7.96 μmol/mmol creatinine; *p* = 0.008, d = 1.14). 1-Methylnicotinamide, the methylated amide of nicotinic acid, was also significantly elevated (Figure 8D) in the urine of the TBM patients (45.30 ± 25.22 μmol/mmol creatinine) when compared to that of the controls (10.61 ± 12.83 μmol/mmol creatinine; p < 0.001, d = 1.82). Both 1-methylnicotinic acid and 1-methylnicotinamide are products of an upregulated kynurenine metabolism (Cazzullo et al., 1976), and, within the tryptophan–nicotinic acid metabolism, 1-methylnicotinamide is an end-product of nicotinamide (vitamin B3) metabolism. Nicotinamide is the precursor of the coenzymes β-nicotinamide adenine dinucleotide (NAD) and nicotinamide adenine dinucleotide phosphate (NADP), which are involved in a variety of enzyme-mediated oxidation and reduction reactions (Li et al., 2006; Zhou et al., 2009). Excess nicotinamide is methylated, oxidized or hydroxylated to 1-methylnicotinamide, nicotinamide-N-oxide or 6- hydroxynicotinamide, respectively, and then 1-methylnicotinamide is further oxidized to the pyridones 1-methyl-2-pyridone-5-carboxamide (2Py) and 1-methyl-4-pyridone-3-carboxamide by aldehyde oxidase (Zhou et al., 2009; Mayneris-Perxachs et al., 2016). 2Py and 1-methylnicotinamide are the major common nicotinamide metabolites found in human urine (Kitamura et al., 2008). A study by Zhou *et al.* (2009) showed that 1-methylnicotinamide clearance was delayed in diabetic patients, suggesting that 1-methylnicotinamide could cause oxidative stress and insulin resistance (discussed further below). 1-Methylnicotinamide may also play a role in the progression of Parkinson’s disease, as a result of a superoxide anion formed by 1-methylnicotinamide via mitochondria (Fukushima et al., 2002; Williams et al., 2005), associated with elevated oxidative stress (Smeitink et al., 2004), and also with major depressive disorders (Zheng et al., 2013); hence, associating increased urinary 1- methylnicotinamide to several neurological and inflammatory conditions. In this study, the significantly elevated concentrations of 1-methylnicotinamide, is most likely due to the increased flux through the kunurenine pathway induced by INF-γ, and generation of reactive oxygen species (ROS) and reactive nitrogen species (RNS)–the consequences of severe oxidative stress and impaired redox status, associated with TB (Reddy et al., 2009; Miric et al., 2013).

### 4.3 Perturbation of amino acid metabolism

The urinary concentrations of tyrosine (Figure 5B) were significantly elevated in the TBM patients (55.09 ± 21.81 μmol/mmol creatinine) compared to the controls (37.2 ± 20.14 μmol/mmol creatinine; *p* = 0.021, d = 0.85). Phenylalanine and tyrosine are aromatic amino acids, synthesized from phosphoenolpyruvate and erythrose 4-phosphate–intermediates of glycolysis and the pentose phosphate pathway, respectively (Ferreira and Teixeira, 2003). Phenylalanine is catabolized into acetoacetic acid and fumaric acid via tyrosine (Komoda and Matsunaga, 2015). Tyrosine is formed by the hydroxylation of phenylalanine in the liver when the intake of tyrosine is low (Litwack, 2018). Both these amino acids serve as precursors for the synthesis of many biologically/neurologically active compounds that are essential for maintaining a variety of biological functions (Han et al., 2019). The neurotransmitters epinephrine and norepinephrine are synthesized from the tyrosine metabolite L-3,4- dihydroxyphenylalanine (L-DOPA). Parkinson’s disease and schizophrenia are thought to be caused by, amongst other factors, a lack of these neurotransmitters (Komoda and Matsunaga, 2015). Hyperphenylalaninemia (HPA), a disorder resulting in levels of phenylalanine that are excessive, is caused by a deficiency of the hepatic phenylalanine-4-hydroxylase (PAH) or its cofactor tetrahydrobiopterin (BH4), and clinically presents with a number of neurological signs and symptoms, such as irritability, hyperkinesis, and severe cognitive deficiency, often associated with TBM (Blau et al., 2014). In TB patients, altered metabolism of phenylalanine and tyrosine was previously observed in a pilot study that compared the urine metabolic profiles of 21 adults [33]; this alteration was confirmed in *M. tb*-infected mice (Shin et al., 2011; Weiner et al., 2012). These studies support our findings of significantly elevated tyrosine in advanced TBM when compared to controls.

Glycine concentrations (Figure 5G) were elevated in the TBM patients (457.19 ± 336.45 μmol/mmol creatinine) when compared to that of the controls (242.51 ± 194.75 μmol/mmol creatinine; *p* = 0.053, d = 0.81), at a practically significant level (d > 0.6). Glycine functions as a neurotransmitter in the brain, allowing neurons to communicate with one another, and subsequently regulates neuronal activity (Shahsavar et al., 2020). Many of the clinical signs and symptoms of TBM, such as low muscle tone, lethargy, seizures, coma, and apnea requiring ventilator support, are associated with glycine accumulation in the brain and neural tissue (DeArmond et al., 2017). Glycine transporters, both astrocytic GlyT1 and presynaptic neuronal GlyT2, are critical for proper glycine recycling at glutamatergic and glycinergic synapses. A number of key studies have shown that microglial activity is modulated by astrocyte-derived glycine and L-serine (Van Den Eynden et al., 2009). Glycine at micromolar concentrations changes the morphology of microglial cells and increases the secretion of nitrogen oxide, superoxide, acid phosphatase, and their metabolic activity when induced by lipopolysaccharides (LPS) (Van Den Eynden et al., 2009). LPS are also well-known for their ability to stimulate the production of proinflammatory cytokines (Jansky et al., 2003; Schildberger et al., 2013), especially IL-6 and IFN-γ, all of which (LPS and the associated cytokines) are also elevated in TB patient blood (Feruglio et al., 2013; Gallucci et al., 2021). Several of the clinical symptoms associated with HPA and an accumulation of glycine in the brain are similar to those clinical symptoms seen in TBM–e.g., altered consciousness and behavior. Thus, the treatment of these amino acid imbalances is a potential basis of therapy to alleviate the symptoms found in TBM cases–something that needs to be tested in a future study.

The branched chain amino acids were all increased in the TBM group–leucine (Figure 5D): 31.67 ± 12.64 μmol/mmol creatinine, isoleucine (Figure 5E): 30.5 ± 31.27 μmol/mmol creatinine and valine (Figure 5F): 31.67 ± 12.64 μmol/mmol creatinine, relative to the controls–leucine: 16.95 ± 7.05 μmol/mmol creatinine; *p* = 0.002, d = 1.49, isoleucine: 10.95 ± 8.08 μmol/mmol creatinine; *p* = 0.052, d = 0.99, valine: 18.88 ± 6.9 μmol/mmol creatinine; *p* < 0.001, d = 1.48. One of the most crucial building blocks for gluconeogenesis are free amino acids, which become elevated when protein synthesis is impaired. These findings are in line with other reports of wasting (cachexia) and malnutrition, also known as “anabolic block,” in TB patients. Here, the term “anabolic block” describes a higher proportion of ingested amino acids being oxidized than being used for protein anabolism (MacAllan et al., 1998; Macallan, 1999; Schwenk and Macallan, 2000). Hence, the significant increase in BCAAs may likely be caused by the increased proteolysis needed to meet the elevated demand for amino acids that are used as fuel sources for energy production during infection (Levin et al., 1983). It is also well recognized that branched chain amino acids (BCAAs) contribute to important metabolic processes in the brain, such as the gluconeogenesis that occurs in activated microglia, as a source of energy during an activated immune response (Sweatt et al., 2004). Furthermore, numerous other pathological conditions are associated with changes to amino acid levels in body fluids (Binici et al., 2023). The level of total amino acids in CSF was measured and examined in a 1981 study on patients who had viral meningitis and TBM. Patients with TBM were found to have significantly higher concentrations of total amino acids, which included leucine, isoleucine and valine (Corston et al., 1981). In a recent investigation of the predictive value of amino acids for bacterial, aseptic, and tuberculous meningitis (n = 41, 41, and 21, respectively), against healthy controls (n = 64), carried out by Binici *et al.* (2023), all BCAAs were found to be significantly increased in TBM subjects, confirming the results. In a ^1^H NMR-based metabolomics study, serum BCAAs were increased in *M. tb*-infected rats, as compared to control rats (Shin et al., 2011) giving support to what we are seeing in this paediatric study population. In conditions of perturbed energy metabolism, as reported by Mason *et al.* (2016b), as analyzed using urine from a similar pediatric TBM cohort, elevated concentrations of hydroxyl acids derived from BCAAs leucine (3-hydroxyisovaleric acid) isoleucine (2-methyl-3-hydroxybutyric acid) and valine (3- hydroxyisobutyric acid) were shown in a putative urinary biosignature. The present study also indicates a comparable metabolic increase in BCAAs to that observed in CSF of children with TBM by Mason *et al.* (2015).

Lastly, lysine, an important amino acid known to facilitate protein synthesis (Chang and Gao, 1995) (Figure 5C) was also significantly increased in the TBM cases (151.55 ± 72.73 μmol/mmol creatinine) when compared to that of the controls (82.59 ± 29.84 μmol/mmol creatinine; *p* = 0.007, d = 1.34). Via it’s already well described neuroprotective and neurotrophic effects, lysine has been shown to enhance neurological function and cerebral blood flow in patients with ischemic stroke (Kondoh et al., 2010). According to reports, intellectual disability and other motor neuron impairment are associated with elevated levels of lysine in the CSF of TBM patients (Shaw et al., 1995a). Additionally, it has been demonstrated to form adducts with various substances, including acrolein-lysine, a marker of lipid peroxidation in pediatric meningitis (Tsukahara et al., 2002). Children with persistent atopic dermatitis have also been shown to exhibit considerably higher urine excretion of acrolein-lysine adducts than do healthy children (unpublished data) (Tsukahara et al., 2002). In this investigation, all TBM patients showed elevated lysine concentration, which is in support of the aforementioned reports, and that of Mason *et al.* (2017), who investigated the utilization of amino acids in CSF, in order to distinguish TBM from healthy controls.

### 4.4 Metabolic burst energy metabolites

In this study, urinary glucose (Figure 7A) was significantly elevated in the TBM patients (114.82 ± 44.86 μmol/mmol creatinine) compared to the controls (61.35 ± 27.63 μmol/mmol creatinine; *p* = 0.001, d = 1.48). Other significantly increased urinary saccharides in TBM included mannose (106.26 ± 62.42 μmol/mmol creatinine) and sucrose (101.48 ± 63.86 μmol/mmol creatinine) when compared to the controls, respectively (17.83 ± 14.63 μmol/mmol creatinine; *p* < 0.001, d = 2.3 and 28.83 ± 12.95 μmol/mmol creatinine; *p* = 0.002, d = 1.89). Myo-inositol (Figure 7A) and pyruvic acid (Figure 7G) were also identified by statistical analysis in this study as being significantly increased in TBM (317.02 ± 145.37 μmol/mmol creatinine and 62.79 ± 34.04 μmol/mmol creatinine, respectively) when compared to the controls (137.99 ± 64.29 μmol/mmol creatinine; *p* = 0.001, d = 1.71 and 27.41 ± 7.49 μmol/mmol creatinine; *p* = 0.004, d = 1.70, respectively).

These increased metabolites–glucose, mannose, sucrose, myo-inositol, pyruvic acid, 3- hydroxyisobutyric acid and 3-hydroxyisovaleric acid–can be linked to metabolic burst, associated with an increased need for energy production in the *M. tb*-infected brain (Mason et al., 2015; Mason, 2017; van Zyl et al., 2020). Glucose is the primary metabolite that is catabolized for neuroenergetics purposes. However, uncontrolled glucose utilization in the brain may subsequently lead to insulin resistance, and transitory glucose oxidation via glucose oxidase during unregulated glucose metabolism. According to a growing body of evidence, chronic neuroinflammatory diseases, such as TBM, are known to be linked to insulin resistance (Isaiah et al., 2020). The oxidation of glucose produces gluconolactone, an inflammatory marker previously observed in pulmonary TB (Preez et al., 2017), when insulin becomes depleted. Gluconolactone is siphoned into the pentose phosphate pathway and leads to elevated levels of hydrogen peroxide (Preez et al., 2017). Various studies (Hampton et al., 1998; Podrez et al., 2000; Klebanoff, 2005; Manyelo et al., 2019) have shown that myeloperoxidase, identified as an immunological marker of TBM, interacts with hydrogen peroxide and triggers a variety of oxidative stress pathways. In our previously published downstream metabolic model of TBM (Isaiah et al., 2020), we predicted end products of myeloperoxidase activation, namely, glutathione sulfonamide and 3- chlorotyrosine. Although we did not detect these two products in the urine of the TBM patients using ^1^H-NMR, it is highly likely that other more sensitive analytical methods (e.g., liquid chromatography coupled to mass spectrometry) may detect these and this could be considered for a later study.

In the brain, lactate has neuroprotective properties, associated with a shuttling neuroenergetic mechanism (Mason, 2017). However, this shuttling role of lactate is compartmentalized within the brain during TBM, and urinary lactate levels do not significantly increase during such times. Systemically, lactate is converted to pyruvate, leading to significantly increased urinary levels of pyruvate in TBM, and an increased conversion of NAD + to NADH. This signals an increased levels of redox and oxidative stress, as previously described in the predictive urinary TBM model by Isaiah *et al.* (2020). This is evidenced when looking at the urinary lactate:pyruvate (Lac:Pyr) ratio, and TBM cases have a significantly lower (*p* = 0.004) Lac-Pyr ratio of 2.43, when compared to that of the controls (6.32). An additional measure of the redox status can be done by determining the ratio of 3-hydroxybutyric acid:acetoacetic acid (3HB:AAA), which is significantly lower (*p* < 0.001) in the TBM cases (1.12), than in the controls (1.93).

3-Hydroxyisobutyric acid, a downstream catabolite of valine metabolism (Ko et al., 1991) (Figure 7D), was significantly increased in the TBM group (38.41 ± 11.78 μmol/mmol creatinine), as well as was the leucine catabolite 3-hydroxyisovaleric acid (Figure 7C, 53. ± 20 μmol/mmol creatinine), when compared to the controls, respectively (24.06 ± 10.32 μmol/mmol creatinine; *p* = 0.001, d = 1.3 and 17.85 ± 8.39 μmol/mmol creatinine; *p* < 0.001, d = 2.48). Both of these hydroxy acids gave further support for the metabolites associated with a perturbed energy state in TBM (Mason et al., 2016b). The accumulation of 3-hydroxyisobutyric acid and 3-hydroxyisovaleric acid, subsequently feeds into the anaplerotic Krebs cycle, for primary energy production. Coincidently, the *M. tb* bacilli also utilize the TCA cycle for their own energy production (Savvi et al., 2008). An additional indicator of perturbed energy metabolism is the elevated myo-inositol detected in the paediatric TBM patients. Myo-inositol is mostly made from glucose (Hauser and Finelli, 1963) and is a key signaling molecule required for immunological responses, such as microglia activation. Myo-inositol has previously been linked to microglia and astrocyte activation, as well as a pathogenic response seen in neurodegenerative illness and neuroinflammation (Pears et al., 2005). Increased CSF myo-inositol in TBM patients has also been reported previously (van Zyl et al., 2020), and this study shows that this is a systemic condition in TBM that is likely linked to uncontrolled glucose utilization.

Mannose is interesting because it is described as being ubiquitous (i.e., it occurs everywhere). It is grouped under energy metabolism here since it is a sugar but mannose can also come from lipoarabinomannan (LAM), one of the key components of the *M. tb* cell wall (Van Toorn et al., 2014). In lab grown *M. tb*, LAM was shown to be composed of D-arabinose (55%–60%), D-mannose (36%–40%), and fatty acyls (1%–3%; palmitate C:16; tuberculostearate (TBSA) C:19:1) (Amin et al., 2021). Further examination of the role of mannose is however still needed.

### 4.5 Disrupted gut microbiota metabolism

Eight of the metabolites listed in Table 2 are urinary indicators of altered metabolism associated with the gut microbiota. A significant increase of 4-hydroxyphenylacetic acid (Figure 6B) was observed in TBM urine (138.78 ± 75.85 μmol/mmol creatinine) compared to the controls (37.45 ± 22 μmol/mmol creatinine; *p* < 0.001, d = 2.07). Elevated levels of o-cresol (Figure 6A) and m-cresol (Figure 6C) (62.15 ± 20.88 μmol/mmol creatinine and 65.57 ± 101.77 μmol/mmol creatinine, respectively) were found in TBM compared to the controls (24.02 ± 8.67 μmol/mmol creatinine; *p* < 0.001, d = 2.58 and 18.5 ± 8.7 μmol/mmol creatinine; *p* = 0.135, d = 0.85, respectively). Other elevated urinary gut microbiota metabolites were formic acid (Figure 6D), methylamine (Figure 6G) and methylguanidine (Figure 6H) (102.66 ± 129.4 μmol/mmol creatinine, 13.56 ± 8.65 μmol/mmol creatinine and 44.7 ± 10.8 μmol/mmol creatinine, respectively), when compared to the controls (31 ± 16.06 μmol/mmol creatinine; *p* = 0.08, d = 0.99, 7.82 ± 3.64 μmol/mmol creatinine; *p* = 0.043, d = 0.93 and 22.03 ± 6.9 μmol/mmol creatinine; *p* < 0.001, d = 2.56). Hippuric acid was identified by statistical analyses as significant based upon the ^1^H NMR spectral data (Table 2). However, the concentration data (Figure 6F) of hippuric acid did not show statistical significance (*p* = 0.096, d = 0.57). Lastly, arabinose (Figure 6E) was identified from the untargeted ^1^H-NMR data as being statistically significant in TBM cases (103.45 ± 40.2 μmol/mmol creatinine) compared to the controls (55.47 ± 17.21 μmol/mmol creatinine; *p* = 0.001, d = 1.67).

Arabinose is another ubiquitous metabolite that is produced by the host and gut microbiota, but is also a component of LAM (Amin et al., 2021). Previous studies have shown arabinose as a proxy for LAM in active TB (De et al., 2015). Hence, it’s significantly elevated concentrations in this pediatric cohort of TBM cases, could be classified as a potential biomarker of *M. tb*–this requires further investigation. Arabinose has also been associated with other diseases. In a case of two autistic brothers, without any known metabolic disease, urinary arabinose concentrations were found to be six times greater than that in healthy children (Shaw et al., 1995b). Furthermore, increased levels of arabinose have been reported to have an inhibitory effect on sucrase (Seri et al., 1996), which could account for the increased levels of urinary sucrose in TBM cases found in this study.

Some organic acids are produced, at least in part, by intestinal gut bacterial metabolism. Clinically, these organic acids can be used as an indirect indicator of dysbiosis (Chapman et al., 2020), and the higher the quantities of these bacterial metabolites in the urine, the greater the bacterial quantities and activity in the gastrointestinal tract (Chapman et al., 2020). Elevated urinary concentrations of gut microbiota metabolites. Chalmers *et al.* (1979) indicated that 4- hydroxyphenylacetic acid is synthesized when amino acids, such as phenylalanine and tyrosine–both elevated in TBM–are metabolized by intestinal bacteria. Tyrosine is degraded to tyramine, and then deaminated and oxidized eventually to form 4-hydroxyphenylacetic acid, which is then excreted unchanged and unconjugated in the urine (van Der Heiden et al., 1971; Fellaman et al., 1977). The latter occurs in children with small-bowel disease or in various bacterial overgrowth syndromes; Chalmers *et al.* (1979) subsequently concluded that urinary 4-hydroxyphenylacetic acid serves as a useful marker for the screening of gut disorders in children (Chalmers et al., 1979). It has also been determined that roughly 50% of cystic fibrosis patients, and some patients with confirmed or suspected TB, have elevated urinary concentrations of 4-hydroxyphenylacetic acid in both adults and children with different types of pulmonary disease (Garrettson et al., 1971; van Der Heiden et al., 1971). The occurrence of 4- hydroxyphenylacetic acid has also been linked to the breakdown of amino acids.

Hippuric acid, also known as benzoylglycine or benzoylamino-acetate, is the glycine conjugate of benzoic acid (Lees et al., 2013). Increased levels of urinary hippuric acid have been linked to several diseases, including dysbiosis. This supports its recognition as a biomarker for microbial changes in the gut (Williams et al., 2010; Chapman et al., 2020). In a gas chromatography–mass spectrometry (GC-MS) metabolomics study conducted on a similar paediatric cohort, Mason *et al.* (2016b) observed a significant increase of hippuric acid in the urine of TBM patients and attributed the observation to a drug-like phase II metabolic response of the host to products generated by the microbiota, indicating a highly active glycine-conjugated GLYAT-biotransformation system in TBM. Hence, the presence of increased urinary hippuric acid in TBM is supported by our previous metabolomics study, in which an alternative analytical platform was used.

Methylamine has been found in numerous tissues and bodily fluids, and it has been hypothesized that methylamine, and other closely similar short-chain aliphatic amines, may be involved in the abnormalities of the central nervous system seen during hepatic and renal disease, particularly when the blood-brain barrier is damaged. When compared to trimethylamine and dimethylamine, there is little information in the literature about the human urinary excretion of methylamine (Mitchell and Zhang, 2001; Pirisino et al., 2005; Guba et al., 2022). Methylguanidine is the immediate precursor to methylamine, thereby linking it to microbial metabolism, however, methylguanidine is also known to be an uremic (neuro)toxin (De Deyn et al., 2001) and an inhibitor of inducible nitric oxide synthase (iNOS), which is known to affect *M. tb* cerebral infection (Poh et al., 2022). Hence, methylguanidine is another metabolite of interest that is potentially produced directly by *M. tb*, and requires further investigation.

Formic acid is a common organic acid used and excreted by bacteria and no specific link has previously been made to TBM, however, formic acid has been associated as a potential biomarker of Alzheimer’s disease, as a byproduct of the metabolism of formaldehyde (Wang et al., 2022). To our knowledge, no literature exists on the two cresols (o-cresol and m-cresol) detected as increased in TBM in this study, in relation to neuropathology.

Thus, these eight increased microbial metabolites originate almost exclusively from altered bacterial metabolism in the gut and are attributed to the severe TBM disease in this study. The occurrence of dysbiosis in TBM is supported by several studies and the results support further investigation of the gut–brain axis in TBM (Isaiah et al., 2020). A major limitation of this study is that we cannot account for treatment given to the TBM cases prior to admission to hospital, nor upon admission, and their effects on the gut microbiota.

### 4.6 Ketoacidosis

Ketoacidosis is a condition defined by increased ketone bodies, such as acetone, acetic acid and acetoacetic acid. Acetone (Figure 8A) and acetic acid (Figure 8B) were significantly elevated in the TBM cases (176.48 ± 179.09 μmol/mmol creatinine and 61.77 ± 16.25 μmol/mmol creatinine, respectively) compared to the controls (26.82 ± 52.59 μmol/mmol creatinine; *p* = 0.014, d = 1.29 and 24.73 ± 8.7 μmol/mmol creatinine; *p* < 0.001, d = 2.97, respectively). However, acetoacetic acid (Figure 8C) in the TBM group was not statistically different from the control group (*p* = 0.111), but were practically significant (d = 0.81).

Ketones are metabolic end-products of fatty acid metabolism that occurs in the liver (Dhillon and Gupta, 2021; Cruzat et al., 2018). During perturbed energy metabolism, or in the absence/diminished availability of carbohydrates, fats become the primary source of energy, and large levels of ketones are produced as a metabolic by-product. Hence, the occurrence of ketoacidosis also supports evidence for insulin resistance, as elevated glucose levels are found in TBM patients. The increased levels of ketone bodies indicate that this elevated glucose is not being used for energy production; instead, fatty acid metabolism has become the major source of energy production in TBM (Blau et al., 2014; Dhillon and Gupta, 2021).

Acetone, which may give the breath of ketotic patients a typical odor, is formed via the non-enzymatic decarboxylation of acetoacetate (Blau et al., 2014). The TBM group’s urinary ketone values in this study are indicative of their poor clinical health. Ketoacidosis also supports the metabolic burst that we described previously. Several ketoacidosis markers were also identified in our previous GC-MS metabolomics study of TBM (Mason et al., 2016b), which included the ketosis markers 2- hydroxybutyric acid, 3-hydroxybutyric acid, 2-methyl-3-hydroxybutyric acid, and acetoacetic acid, in the urine of the TBM patients.

### 4.7 Increased nitrogen excretion

Urea was identified in the statistical analyses of the raw data as being significantly increased in the urine of the TBM cases compared to the controls. Increased urea is irrevocably linked to elevated nitrogen excretion. Urea in a ^1^H NMR spectrum presents as a very distinctive broad singlet near to the suppressed water signal. Owing to the width of the urea signal in the ^1^H NMR spectra, and the known influence of water suppression on the urea peak, accurate quantification of urea was not possible.

N-Acetylglutamine (Figure 8F), another metabolite of increased nitrogen excretion, was significantly elevated in the TBM patients (335.45 ± 184.47 μmol/mmol creatinine) compared to the controls (102.43 ± 25.98 μmol/mmol creatinine; *p* ≤ 0.001, d = 2.21). N-Acetylglutamine is an amino acid derivative, and the downstream metabolite of D-glutamine and D-glutamate metabolism (Zhou et al., 2018). N- acetylglutamine is used as a neurotransmitter in the brain, and perturbed N-acetylglutamine can be linked to altered neuronal stability and/or function. In a study that conducted RNA-sequencing on whole blood and CSF of ventricular and lumbar punctures of paediatric patients treated for TBM, Rohlwink *et al.* (2019) indicated immune responses and neural excitotoxicity which depicts the immunological events that occurs in the brain. Of the 389 differentially expressed genes, 45 genes are mainly associated with glutamate excitotoxicity, key excitatory neurotransmitter in learning and memory also, mediator associated with several neurological diseases (Rohlwink et al., 2019). N-Acetylglutamine is also classified as a uremic toxin and increased urinary levels indicate severe neurological complications (typical in TBM), advanced catabolism and/or possible kidney damage. Glutamine supplementation is a well-known strategy to treat some critically ill patients, such as prematurely born infants (Darmaun et al., 2018), and could be considered in paediatric TBM.

### 4.8 Urinary metabolic map of TBM

**FIGURE 9:**
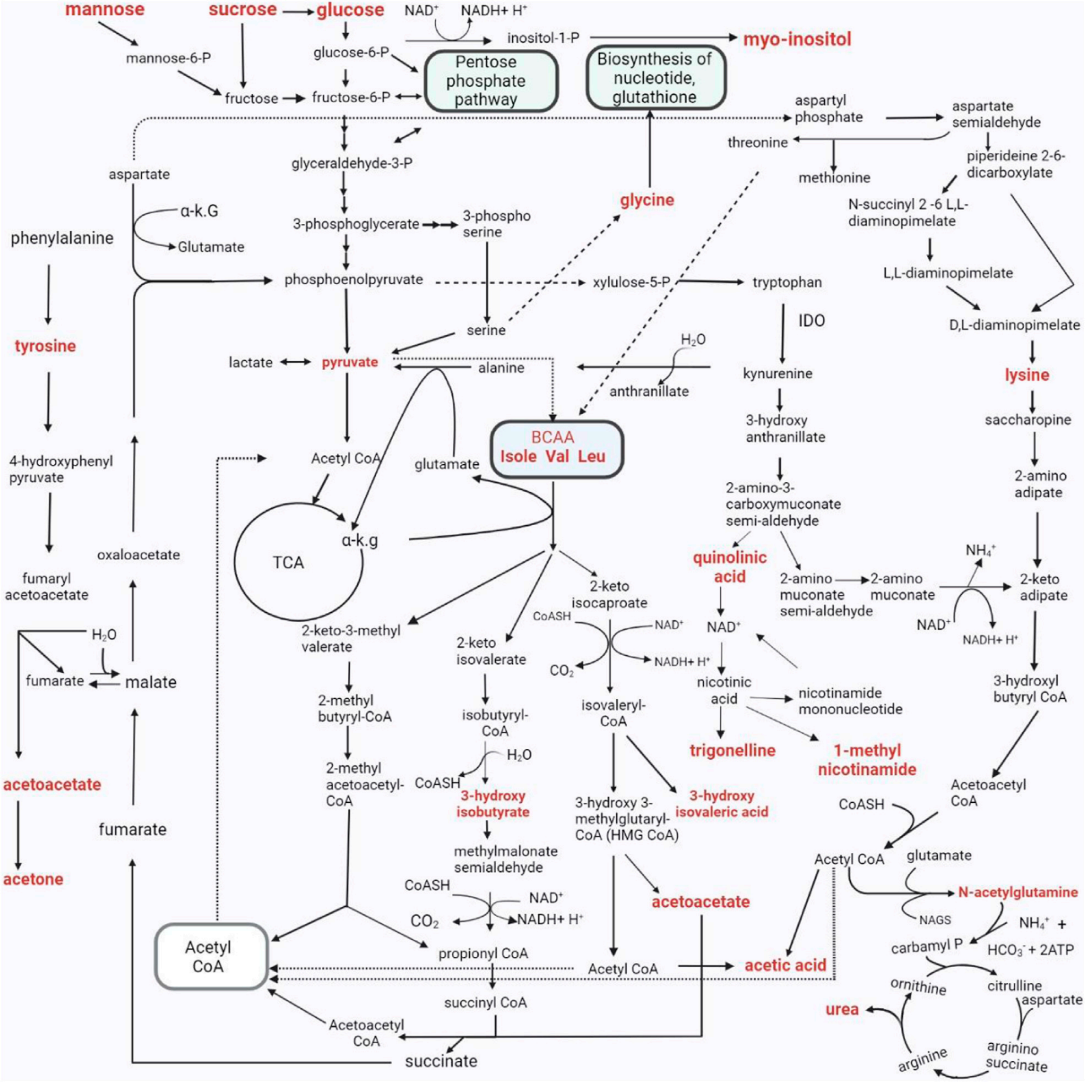
The metabolic pathways characterizing the changed urinary metabolic profiles in TBM stage 3 patients. The metabolites in bold denote those detected in significantly elevated concentrations in the TBM stage 3 patients when compared to the controls. As indicated in the pathway, P–phosphate; α K.G–alpha ketoglutarate; IDO - Indoleamine 2, 3-dioxygenase; Isole–isoleucine; Val–valine; Leu–leucine; CoA, CoASH–coenzyme A; TCA - tricarboxylic acid cycle; NH_4_^+^–ammonium; HCO_3_^−^–bicarbonate; ATP–adenosine triphosphate; NAD–nicotinamide adenine dinucleotide; BCAA–branched-chain amino acids.; H_2_O–water; NAGS–N-acetyl glutamate synthase.

We took all of the above described metabolic pathways characterizing the urinary profiles of advanced hospitalized TBM paediatric cases, and connected them into a metabolic map (Figure 9). Figure 9 illustrates how all these metabolic pathways connect with each other, providing new connections (information) of metabolic pathways, and their complexity, in advanced TBM in paediatric patients. With this new information, we offer new insights and approaches into future TBM research.

### 4.9 Limitations of the study and TB medications

The limitations in this study included: 1) a small sample size (no *a priori* sample size considerations were factored in because the samples were retrospectively selected from a previous study), 2) no validation sample cohort, 3) there was no information available on the timing and type of treatment regime administered to the undiagnosed TBM patients at the referral healthcare centers, before being taken to the hospital, 4) the controls were heterogenous and, due to ethical restrictions, the only information available for the controls were that they were pediatric patients without meningitis, no neurological symptoms, and from the same geographical region as the TBM patients. Upon inspection of the ^1^H-NMR spectra, it was found that pyrazinamide (PZA), isoniazid (IZA) and ethionamide were detected in all the time zero stage three (T0) TBM cases urine samples, except for 2 cases (103 and 114) which had no IZA and no ethionamide. Rifampicin and its two metabolites–3- formylrifampicin and 25-deacetyl-rifampicin, were not detectable in the ^1^H-NMR spectra in any of the urine samples. The inability to see rifampicin and its two known urinary metabolites could be that either the rifampicin has been detoxified in the body into a form that we do not know of, or, more likely, the NMR analytical platform lacks the sensitivity (i.e., below the detection limit of the NMR).

## 5 Conclusion

We consider the significant metabolites identified in this study as being the best urinary metabolic characterization of TBM, to date. The precise outcome of this study indicated that TBM under initial treatment could be well differentiated from controls, based upon urinary metabolic profiles analyzed by ^1^H NMR spectroscopy. These results support metabolomics data produced in our previous GC-MS metabolomics study conducted using a similar paediatric cohort. Thus, these results support the use of ^1^H NMR metabolomics in characterizing TBM, and in the pursuit of non-invasive metabolic markers of TBM to aid in the early differential diagnosis and subsequent treatment of TBM–something that is greatly needed in the paediatric population, the patient group most at risk of mortality.

Moreover, several clinical aspects of the studied cohort of advanced TBM were inferred from the urinary metabolomics data, which need to be confirmed from blood samples collected from TBM cases. The findings related to -illness induced-malnutrition can have therapeutic impact. Malnutrition can be subdivided in deficiency of micronutrients (e.g., vitamins) and macronutrients (e.g., proteins). We found (a) deficient vitamin B3 status expressed by increased urinary 1-methylnicotinamide–upregulated utilization of vitamin B3 from increased tryptophan catabolism, and increased urinary trigonelline; (b) increased nitrogen excretion, which reflects the elevated catabolic state of patients and their related malnutrition. For instance, shortage of the semi essential amino acid glutamine can lead to immune dysfunction (Cruzat et al., 2018). Thus, it is our recommendation that the nutritional status of TBM cases should be assessed with comparison made to pulmonary TB cases or controls, in order to determine whether the findings are specific to TB or TBM. Potentially, additional supplementation of vitamin B and glutamine may be a consideration in the nutritional component of the future overall management of TBM.

## Data Availability

The original contributions presented in the study are included in the article/Supplementary Material, further inquiries can be directed to the corresponding author.
