# Supplementary material for: Urinary metabolic characterization of advanced tuberculous meningitis cases in a South African paediatric population
Source: Front Mol Biosci. 2024 Mar 15;11:1253983. doi: 10.3389/fmolb.2024.1253983 (PMC10978807; doi:10.3389/fmolb.2024.1253983)
Supplement: Supplementary file 1 [file DataSheet1.DOCX]

Supplementary Information:

**Urinary metabolic characterization of advanced tuberculous meningitis cases in a South African paediatric population**

**Simon Isaiah1, Du Toit Loots1, Mari Van Reenen1, Regan Solomons2, Sabine L. van Elsland3, AM Tutu van Furth4, Martijn van der Kuip4, Shayne Mason1***

#
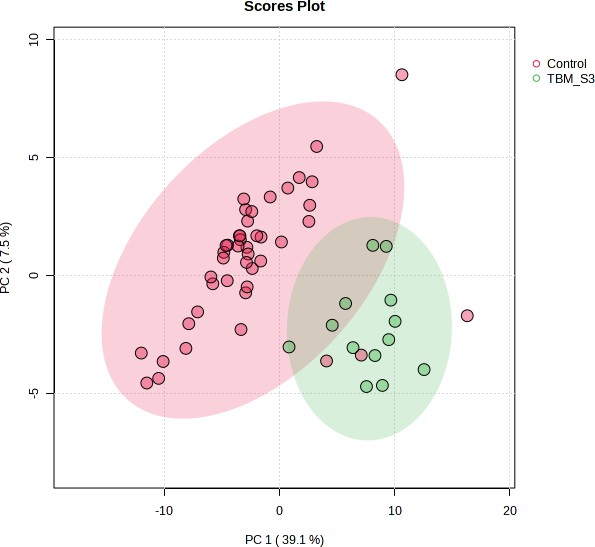


**Fig. S1.** Four outliers identified in control group using PCA with 95% CI.

In review


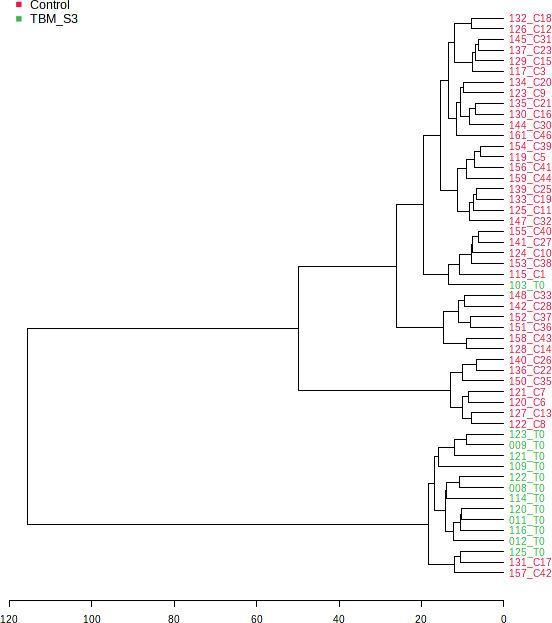


**Fig. S2.** Hierarchical cluster analysis results shown as dendrograms (Euclidean distance measure, and clustering algorithm using Ward’s method). All cases of controls (n = 40, in red) and TBM cases (n = 13, in green).

In review

| Metabolite annotation | Bin | VIP  Comp. 1 | VIP  Comp. 2 | p-value  FDR | Fold Change |
| --- | --- | --- | --- | --- | --- |
| Isoleucine | 0.93 | 0.99 | 1.05 | <0.00001 | 2.23 |
| Isoleucine/Leucine | 0.95 | 1.22 | 1.23 | <0.00001 | 2.65 |
| Leucine | 0.97 | 1.28 | 1.24 | <0.00001 | 2.67 |
| Valine | 0.99 | 1.13 | 1.01 | 0.0006 | 3.30 |
| Isoleucine/Valine | 1.01 | 1.21 | 1.07 | 0.0001 | 3.91 |
| Valine | 1.03 | 1.08 | 1.06 | 0.0001 | 3.62 |
| Valine | 1.05 | 1.00 | 0.91 | 0.0006 | 2.71 |
| 3-Hydroxyisobutyric acid | 1.07 | 1.30 | 1.17 | 0.0001 | 2.97 |
|  | 1.09 | 1.49 | 1.44 | <0.00001 | 3.78 |
| 1,2-Propanediol | 1.13 | 1.86 | 1.65 | <0.00001 | 7.51 |
|  | 1.15 | 2.24 | 1.99 | <0.00001 | 18.81 |
|  | 1.17 | 0.91 | 0.94 | 0.0005 | 2.13 |
| 3-Hydroxyisovaleric acid | 1.27 | 1.38 | 1.24 | <0.00001 | 2.92 |
| 1.29 | 1.29 | 1.20 | 1.09 | <0.00001 | 2.63 |
| 1.41 | 1.41 | 1.09 | 1.18 | <0.00001 | 2.34 |
| 1.43 | 1.43 | 1.26 | 1.20 | <0.00001 | 2.96 |
| 1.45 | 1.45 | 1.22 | 1.29 | <0.00001 | 2.74 |
| 1.51 | 1.51 | 1.38 | 1.31 | <0.00001 | 3.21 |
| 1.55 | 1.55 | 1.50 | 1.34 | <0.00001 | 3.83 |
| 1.57 | 1.57 | 1.48 | 1.32 | <0.00001 | 3.75 |
| 1.61 | 1.61 | 1.16 | 1.14 | <0.00001 | 2.52 |
| Lysine | 1.75 | 1.06 | 0.98 | <0.00001 | 2.39 |
|  | 1.77 | 0.97 | 0.89 | <0.00001 | 2.13 |
| Acetatic acid | 1.93 | 1.09 | 0.97 | <0.00001 | 2.05 |
| N-Acetylglutamine | 2.07 | 1.42 | 1.33 | <0.00001 | 3.24 |
| Acetaminophen | 2.15 | 1.76 | 1.57 | <0.00001 | 5.61 |
| 2.19 | 2.19 | 1.02 | 1.12 | 0.0001 | 2.46 |
| o-Cresol | 2.21 | 1.32 | 1.19 | <0.00001 | 3.08 |
| Acetone | 2.23 | 1.12 | 1.00 | <0.00001 | 2.98 |
| Acetoacetic acid | 2.29 | 0.81 | 0.78 | 0.0005 | 2.13 |
| m-Cresol | 2.31 | 0.94 | 0.94 | <0.00001 | 2.08 |
| Aspirin | 2.33 | 1.53 | 1.41 | <0.00001 | 6.17 |
| Pyruvic acid | 2.39 | 1.10 | 0.98 | <0.00001 | 2.13 |
| 2.59 | 2.59 | 1.07 | 0.97 | 0.0004 | 2.68 |
| Methylamine | 2.61 | 1.30 | 1.17 | <0.00001 | 3.10 |
| 2.75 | 2.75 | 1.26 | 1.17 | <0.00001 | 2.61 |
| 2.77 | 2.77 | 1.40 | 1.28 | <0.00001 | 3.07 |
| 2.79 | 2.79 | 1.15 | 1.14 | <0.00001 | 2.37 |
| 2.81 | 2.81 | 2.01 | 1.84 | <0.00001 | 6.58 |
| Methylguanodine | 2.83 | 1.05 | 1.13 | <0.00001 | 2.22 |
| 2.85 | 2.85 | 1.89 | 1.73 | <0.00001 | 6.38 |
| 2.87 | 2.87 | 1.34 | 1.21 | <0.00001 | 2.94 |
| 2.89 | 2.89 | 1.48 | 1.34 | <0.00001 | 3.19 |
| 2.95 | 2.95 | 1.11 | 1.00 | <0.00001 | 3.08 |
| 2.97 | 2.97 | 1.57 | 1.46 | <0.00001 | 3.47 |
| myo-Inositol | 3.31 | 1.20 | 1.10 | <0.00001 | 4.00 |
| 3.35 | 3.35 | 0.93 | 1.06 | <0.00001 | 2.05 |
| 3.37 | 3.37 | 1.16 | 1.12 | <0.00001 | 2.48 |
| Glucose | 3.41 | 1.21 | 1.17 | <0.00001 | 2.75 |
| Glucose/1,2-Propanediol | 3.45 | 1.68 | 1.55 | <0.00001 | 4.77 |
| Glucose | 3.47 | 1.47 | 1.31 | <0.00001 | 4.13 |
|  | 3.49 | 1.14 | 1.08 | <0.00001 | 2.46 |
|  | 3.51 | 1.18 | 1.07 | <0.00001 | 2.34 |
| myo-Inositol/Glucose | 3.53 | 1.37 | 1.22 | <0.00001 | 2.98 |
| 1,2-Propanediol | 3.55 | 1.70 | 1.52 | <0.00001 | 5.77 |
| Glycine | 3.57 | 1.12 | 1.00 | <0.00001 | 2.47 |
| 3.59 | 3.59 | 1.31 | 1.17 | <0.00001 | 2.83 |
| myo-Inositol | 3.61 | 1.17 | 1.06 | <0.00001 | 2.27 |
| Arabinose | 3.69 | 1.12 | 1.00 | <0.00001 | 2.23 |
| Glucose | 3.71 | 1.26 | 1.12 | <0.00001 | 2.69 |
|  | 3.73 | 1.11 | 0.99 | <0.00001 | 2.27 |
|  | 3.75 | 1.10 | 0.98 | <0.00001 | 2.12 |
|  | 3.83 | 1.20 | 1.08 | <0.00001 | 2.47 |
|  | 3.87 | 1.21 | 1.08 | <0.00001 | 2.52 |
|  | 3.89 | 1.44 | 1.28 | <0.00001 | 3.69 |
| Arabinose/Glucose | 3.91 | 1.28 | 1.14 | <0.00001 | 2.84 |
| Sucrose | 4.23 | 1.53 | 1.36 | <0.00001 | 3.58 |
| 4.25 | 4.25 | 1.51 | 1.35 | <0.00001 | 3.19 |
| 4.27 | 4.27 | 1.10 | 1.10 | <0.00001 | 2.32 |
| 4.29 | 4.29 | 1.32 | 1.23 | <0.00001 | 3.33 |
| 4.31 | 4.31 | 1.17 | 1.13 | <0.00001 | 2.47 |
| 4.33 | 4.33 | 1.66 | 1.47 | <0.00001 | 3.97 |
| 4.35 | 4.35 | 1.20 | 1.26 | <0.00001 | 2.28 |
| 4.37 | 4.37 | 1.47 | 1.35 | <0.00001 | 3.11 |
| 4.43 | 4.43 | 1.18 | 1.05 | <0.00001 | 2.23 |
| Trigonelline | 4.45 | 1.18 | 1.06 | <0.00001 | 2.41 |
| 4.47 | 4.47 | 1.72 | 1.63 | <0.00001 | 4.45 |
| 1-Methylnicotinamide | 4.49 | 1.98 | 1.94 | <0.00001 | 6.43 |
| Arabinose | 4.51 | 1.13 | 1.24 | <0.00001 | 3.41 |
|  | 4.53 | 1.63 | 1.72 | <0.00001 | 5.67 |
| 4.57 | 4.57 | 1.61 | 1.59 | <0.00001 | 5.47 |
| 4.59 | 4.59 | 1.41 | 1.49 | <0.00001 | 4.73 |
| 4.65 | 4.65 | 0.83 | 0.75 | 0.0031 | 2.54 |
| 4.67 | 4.67 | 0.70 | 0.69 | 0.0038 | 2.44 |
| Mannose | 5.19 | 1.61 | 1.65 | <0.00001 | 5.28 |
| 5.21 | 5.21 | 1.12 | 1.04 | 0.0002 | 2.02 |
| Arabinose/Glucose | 5.25 | 1.81 | 1.77 | <0.00001 | 5.06 |
| Sucrose | 5.41 | 1.10 | 1.09 | <0.00001 | 3.90 |
|  | 5.43 | 0.63 | 0.66 | 0.0036 | 2.25 |
| Urea | 5.51 | 0.48 | 0.49 | 0.0202 | 2.13 |
|  | 5.55 | 0.78 | 0.75 | 0.0006 | 2.61 |
|  | 5.61 | 0.74 | 0.66 | 0.0056 | 2.37 |
|  | 5.63 | 0.77 | 0.69 | 0.0074 | 2.24 |
|  | 5.65 | 0.79 | 0.71 | 0.0141 | 2.01 |
|  | 5.97 | 0.82 | 0.73 | 0.0063 | 2.28 |
| 6.67 | 6.67 | 0.60 | 0.68 | 0.0036 | 2.01 |
| 6.71 | 6.71 | 0.78 | 0.72 | 0.0006 | 2.58 |
| 6.85 | 6.85 | 1.11 | 1.10 | <0.00001 | 3.86 |
| 4-Hydroxyphenylacetic acid | 6.87 | 1.77 | 1.63 | <0.00001 | 6.04 |
| Tyrosine | 6.89 | 0.87 | 0.82 | 0.0106 | 2.57 |
|  | 6.91 | 1.04 | 0.99 | 0.0006 | 2.80 |
| 7.01 | 7.01 | 0.35 | 0.77 | 0.0436 | 0.43 |
| 7.09 | 7.09 | 0.73 | 0.93 | 0.0287 | 2.32 |
| 4-Hydroxyphenylacetic acid | 7.17 | 1.46 | 1.32 | <0.00001 | 4.06 |
| 7.51 | 7.51 | 0.72 | 0.69 | 0.0327 | 2.93 |
| 7.59 | 7.59 | 1.22 | 1.11 | <0.00001 | 4.24 |
| 7.67 | 7.67 | 0.74 | 0.66 | 0.0238 | 2.29 |
| 7.69 | 7.69 | 1.52 | 1.37 | <0.00001 | 3.37 |
| Isoniazid | 7.73 | 1.65 | 1.48 | <0.00001 | 5.82 |
| Isonicotinic acid | 7.75 | 2.42 | 2.29 | <0.00001 | 15.61 |
| 2-Pyridin-4-formidoacetic acid | 7.77 | 1.97 | 1.87 | <0.00001 | 8.43 |
| Acetylisoniazid | 7.81 | 2.64 | 2.47 | <0.00001 | 20.51 |
| Hippuric acid | 7.87 | 1.29 | 1.21 | 0.0004 | 5.67 |
| 7.89 | 7.89 | 1.65 | 1.63 | <0.00001 | 5.53 |
| 7.91 | 7.91 | 1.43 | 1.27 | <0.00001 | 4.80 |
| 7.93 | 7.93 | 1.06 | 0.94 | 0.0036 | 2.25 |
| 7.95 | 7.95 | 1.51 | 1.49 | <0.00001 | 5.85 |
| Quinolinic acid | 8.01 | 1.22 | 1.08 | 0.0003 | 4.46 |
|  | 8.03 | 1.51 | 1.37 | 0.0001 | 5.31 |
|  | 8.05 | 1.04 | 0.93 | 0.0031 | 5.00 |
| 5-Hydroxy-2-pyrazine carboxylic acid | 8.07 | 0.51 | 0.51 | 0.0202 | 2.90 |
| 1-Methylnicotinamide | 8.17 | 1.30 | 1.17 | 0.0003 | 6.04 |
| 8.33 | 8.33 | 1.72 | 1.78 | <0.00001 | 9.95 |
| 8.35 | 8.35 | 0.86 | 0.78 | 0.0090 | 4.26 |
| Quinolinic acid/Formic acid | 8.47 | 1.21 | 1.11 | <0.00001 | 3.74 |
| 8.49 | 8.49 | 0.61 | 0.57 | 0.0036 | 2.04 |
| Isonicotinic acid | 8.61 | 1.60 | 1.55 | <0.00001 | 7.17 |
|  | 8.63 | 2.30 | 2.22 | <0.00001 | 14.18 |
| 8.67 | 8.67 | 0.63 | 0.58 | 0.0036 | 2.27 |
| Pyrazine carboxylic acid | 8.69 | 2.45 | 2.40 | <0.00001 | 13.68 |
|  | 8.71 | 2.10 | 2.02 | <0.00001 | 8.16 |
| Acetylisoniazid | 8.73 | 2.57 | 2.47 | <0.00001 | 21.19 |
| 8.75 | 8.75 | 1.94 | 1.74 | <0.00001 | 9.57 |
| Pyrazine carboxamide | 8.77 | 1.53 | 1.37 | <0.00001 | 5.69 |
|  | 8.81 | 0.81 | 0.72 | 0.0011 | 2.50 |
|  | 8.83 | 1.29 | 1.16 | <0.00001 | 4.25 |
| Pyrazine carboxylic acid | 9.09 | 2.22 | 2.19 | <0.00001 | 10.55 |
| Pyrazine carboxamide | 9.19 | 1.20 | 1.08 | <0.00001 | 3.56 |

**Table S1.** Quantitative statistical data of the 137 ^1^H-NMR spectral bins, with metabolite annotations, that were identified as important based upon the rule: VIP comp 1 and comp 2 > 1.0 OR p-value FDR ≤ 0.05 and absolute fold change ≥ 2.0.

In review
